# Radix-4 CORDIC algorithm based low-latency and hardware efficient VLSI architecture for *N*th root and *N*th power computations

**DOI:** 10.1038/s41598-023-47890-3

**Published:** 2023-11-27

**Authors:** Ankur Changela, Yogesh Kumar, Marcin Woźniak, Jana Shafi, Muhammad Fazal Ijaz

**Affiliations:** 1 Department of Information and Communication Technology, School of Technology, Pandit Deendayal Energy University, Gandhinagar, Gujarat India; 2 Department of Computer Science and Engineering, School of Technology, Pandit Deendayal Energy University, Gandhinagar, Gujarat India; 3 Faculty of Applied Mathematics, Silesian University of Technology, Kaszubska 23, 44100 Gliwice, Poland; 4 Department of Computer Science, College of Arts and Science, Prince Sattam bin Abdul Aziz University, Wadi Ad-Dawasir, 11991 Saudi Arabia; 5 School of IT and Engineering, Melbourne Institute of Technology, Melbourne, 3000 Australia

**Keywords:** Applied mathematics, Computational science, Computer science

## Abstract

In this article, a low-complexity VLSI architecture based on a radix-4 hyperbolic COordinate Rotion DIgital Computer (CORDIC) is proposed to compute the $N{{\rm th}}$ root and $N{{\rm th}}$ power of a fixed-point number. The most recent techniques use the radix-2 CORDIC algorithm to compute the root and power. The high computation latency of radix-2 CORDIC is the primary concern for the designers. $N{{\rm th}}$ root and $N{{\rm th}}$ power computations are divided into three phases, and each phase is performed by a different class of the proposed modified radix-4 CORDIC algorithms in the proposed architecture. Although radix-4 CORDIC can converge faster with fewer recurrences, it demands more hardware resources and computational steps due to its intricate angle selection logic and variable scale factor. We have employed the modified radix-4 hyperbolic vectoring (R4HV) CORDIC to compute logarithms, radix-4 linear vectoring (R4LV) to perform division, and the modified scaling-free radix-4 hyperbolic rotation (R4HR) CORDIC to compute exponential. The criteria to select the amount of rotation in R4HV CORDIC is complicated and depends on the coordinates $X^j$ and $Y^j$ of the rotating vector. In the proposed modified R4HV CORDIC, we have derived the simple selection criteria based on the fact that the inputs to R4HV CORDIC are related. The proposed criteria only depend on the coordinate $Y^j$ that reduces the hardware complexity of the R4HV CORDIC. The R4HR CORDIC shows the complex scale factor, and compensation of such scale factor necessitates the complex hardware. The complexity of R4HR CORDIC is reduced by pre-computing the scale factor for initial iterations and by employing scaling-free rotations for later iterations. Quantitative hardware analysis suggests better hardware utilization than the recent approaches. The proposed architecture is implemented on a Virtex-6 FPGA, and FPGA implementation demonstrates $19\%$ less hardware utilization with better error performance than the approach with the radix-2 CORDIC algorithm.

## Introduction

The computation of $N{{\rm th}}$ roots and powers is a part of various real-time applications across different fields. Real-time applications in the fields of robotics, 3-D graphics rendering, image and video processing, real-time object recognition, and signal processing, to mention a few, require the computation of the root and power^1–5^. The power-law (Gamma) transformation is a popular image enhancement technique, and part of real-time image and video processing applications. The power-law transform can be characterized using equation $p=c\times q^{\gamma }$, where *q* and *p* represent the input and output pixel value, *c* is constant and $\gamma$ represents the enhancement factor^6^. A $N{{\rm th}}$ root and power are especially helpful in physics and engineering, where calculations involving growth, decay, and change rates are frequent. $N{{\rm th}}$ roots are also used in computer science and cryptography, which helps to create safe algorithms and effective data processing methods. The basis for exponential growth and decay functions, polynomial expressions, and the idea of dimensions are provided by $N{{\rm th}}$ power, which is crucial in algebra, calculus, and geometry. In scientific modelling, $N{{\rm th}}$ powers frequently represent processes ranging from population expansion to radioactive decay.

For real-time applications, the speed of processing incoming data is crucial. Achieving the necessary speed and power performance often requires dedicated hardware, as software alone may not be adequate to deliver the desired performance. Many researchers have proposed a variety of methods that perform multiple square roots and cube roots^7^. The classical approach to computing these roots is the Newton-Raphson (NR) method^8–10^. The $N{{\rm th}}$ root of an integer may be calculated using the Newton-Raphson method, a potent numerical approach for approximating equation solutions. This approach iteratively improves a first guess until it converges to a more precise answer. The demerit of the NR method is that the precision relies on the initial guess, and it requires significant resources as it repeatedly performs multiplication. The trade-off between computational complexity and memory consumption for various NR methods is presented in^11^.

A popular method for carrying out several mathematical operations, including the computation of $N{{\rm th}}$ roots and $N{{\rm th}}$ powers, is the CORDIC algorithm^12–15^. CORDIC is a versatile method for numerical computing since it was first designed to do efficient trigonometric calculations and has since been modified to handle a variety of tasks. Various complex and scaling-free CORDIC approaches were also presented to overcome the various drawbacks^16–18^. The CORDIC is used to carry out a wide variety of applications from eigenvalue decomposition^19–21^ to many real-time DSP applications^22–24^. In research^25^, the CORDIC-based efficient way to calculate the $N{{\rm th}}$ roots and $N{{\rm th}}$ powers is demonstrated which is based on logarithm and exponential. Operations like logarithm and exponential can be efficiently carried out by the CORDIC algorithm. Iterative computations are used by the CORDIC method to estimate the intended outcome. The algorithm may need more iterations, which would increase the computing time, depending on the degree of accuracy required. The CORDIC algorithm is best suited for computations within a specific range, and may not be suitable for many real-time applications.

High-radix CORDIC allows for executing multiple repetitions in parallel, resulting in the reduced number of repetitions directed to achieve the desired accuracy. With each iteration, multiple computations can be carried out simultaneously, leading to faster convergence. By executing multiple iterations in parallel, the algorithm can achieve higher throughput and more efficient resource utilization. This can result in fewer computation times and hardware complexity, making it suitable for hardware acceleration. In this article, we have demonstrated a radix-4 CORDIC-based hardware efficient approach to achieve root and power calculations.

## Related work

This section covers the typical CORDIC method-based architecture and the various radix-2 CORDIC algorithm classes used to calculate the root and power. The standard CORDIC’s input range is its restriction. For various operating modes, the real input range of the typical CORDIC algorithm is addressed. This section also discusses the two strategies for handling a narrow convergence range.

**Table 1:** Various classes of CORDIC algorithm and their output.

Class	Output	Convergence criteria	Function to be evaluated
HR	$$\begin{array}{l}X^N=K_h\ (X^0\ \cosh Z^0-Y^0\ \sinh Z^0)\\ Y^N=K_h (X^0 sinh Z^0+Y^0 coshZ^0)\\ Z^N\approx 0 \end{array}$$	$\vert Z^0 \vert \le 1.1182$	$$\begin{array}{l} \hbox {Initial Value: } X^0 =\dfrac{1}{K_h},\,Y^0 =0\hbox {, and }Z^0=\theta \\ \hbox {Exponential: }e^\theta = \dfrac{ X^N + Y^N }{2} = \dfrac{\cosh \theta + \sinh \theta }{2} \end{array}$$
LR	$$\begin{array}{l}X^N = X^0 \\ Y^N=Y^0+X^0Z^0 \\ Z^N \approx 0 \end{array}$$	–	$$\begin{array}{l} \hbox {Initial Value: } X^0 =\alpha , Y^0 =0\hbox {, and }Z^0=\beta \\ \hbox {Multiplication: } Y^N= \alpha \beta \end{array}$$
LV	$$\begin{array}{l} X^N = X^0 \\ Y^N \approx 0 \\ Z^N =Z^0+ \dfrac{ Y^0 }{ X^0} \end{array}$$	$\vert \dfrac{Y^0}{X^0} \vert \le 2$	$$\begin{array}{l} \hbox {Initial Value: } X^0 =\alpha ,\, Y^0 =\beta \hbox { , and } Z^0=0 \\ \hbox {Division: } Z^N= \dfrac{\beta }{\alpha } \end{array}$$
HV	$$\begin{array}{l} X^N=K_h \sqrt{(X^0)^2 - (Y^0)^2} \\ Y^N \approx 0 \\ Z^N =Z^0+ \tanh ^{-1}\left( \dfrac{ Y^0 }{ X^0}\right) \end{array}$$	$$\begin{array}{l} \vert \tanh ^{-1}\left( \dfrac{Y^0}{X^0} \right) \vert \\ \le 1.1182 \end{array}$$	$$\begin{array}{l} \hbox {Intial Value: } X^0 =\alpha , Y^0=\beta \hbox {, and }Z^0=0 \\ \hbox {Inverse Hyperbolic: } Z^N= \tanh ^{-1}\left( \dfrac{\beta }{\alpha } \right) \end{array}$$

*HR*: Hyperbolic rotation,* LR*: Linear rotation,* LV*: Linear vectoring, and* HV*: Hyperbolic vectoring

### Radix-2 CORDIC algorithm

The CORDIC is well known for the calculation of complex mathematical functions using very simple hardware. The various classes of the CORDIC algorithm can be created by choosing an appropriate operating mode (vectoring or rotation) and coordinate system (circular, hyperbolic, or linear). The generalized form is illustrated below.

1 $$\begin{aligned} \begin{bmatrix} X^{j+1} \\ Y^{j+1} \end{bmatrix}&= \begin{bmatrix} 1 &{} -q\alpha ^{j}2^{-j} \\ \alpha ^{j}2^{-j} &{} 1 \end{bmatrix} \begin{bmatrix} X^{j} \\ Y^{j} \end{bmatrix} \nonumber \\ Z^{j+1}&= Z^j - \alpha ^{j} \beta ^{j} \end{aligned}$$

where parameter q, $\beta ^{j}$, and $\alpha ^{j}$ indicate the coordinate system, rotation angle, and direction of the micro-rotation, respectively. By choosing the appropriate value of q and $\alpha ^{j}$, six different classes of the CORDIC algorithm can be generated. For the root and power calculations, circular CORDIC is not required and they are not discussed here. The output of the other classes of the CORDIC algorithm after convergence and the initial values used to achieve the output are listed in Table 1. The coordinate equations for HV-CORDIC and HR-CORDIC can be derived from Eq. (1) by taking $q=-1$. For hyperbolic CORDIC to achieve convergence, iterations with indexes $j=\left( 3n+1\right) =4,\ 13,\ 40,\ldots$ need to be repeated. The convergence criteria of HV-CORDIC are illustrated as follows:

2 $$\begin{aligned} \tanh ^{-1}\left( \dfrac{Y^0}{X^0} \right) \le \theta _{max} = \sum _{j=1}^{n} = 1.1182 \end{aligned}$$

Similarly, the convergence criterion of HR-CORDIC is $\vert Z^0 \vert \le 1.1182$. Among all six classes of the CORDIC algorithm, LV and LR have the simplest convergence, and they are very similar to the shift and accumulate architecture of a conventional multiplier. The aforementioned hyperbolic computation augments the coordinates by $K_h = \prod _{j=1}^{n}\sqrt{\left( 1-2^{-2j}\right) }$. However, this scale factor can be ignored for HV-CORDIC, as only the value of the Z coordinate is required after the convergence. For HR-CORDIC, the scale factor can be compensated by choosing the initial value of the X coordinate as $X_0=\dfrac{1}{K_h}$. The implementation of root and power computations using these classes of the CORDIC algorithm is discussed next.

### Conventional architecture to compute $N{{\rm th}}$ root and $N{{\rm th}}$ power

The conventional way to determine root and power is based on the following illustrations:

3 $$\begin{aligned} P^{\dfrac{1}{N}}&= e^{\dfrac{\ln {P}}{N}} \nonumber \\ P^N&=e^{N\ln P} \end{aligned}$$

**Figure 1:**
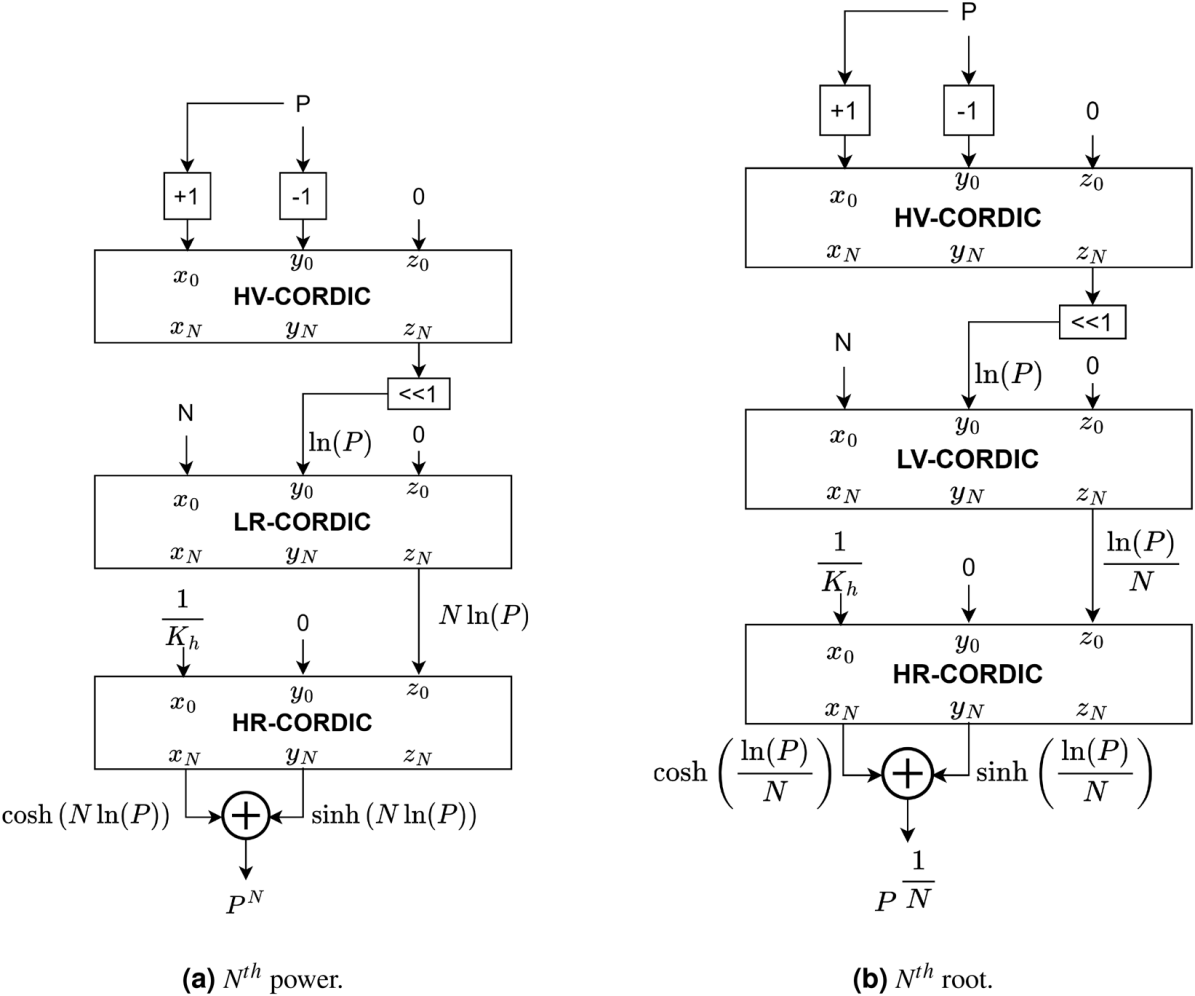
Standard approach to compute root and power.

A specific CORDIC method may be used to implement the logarithm and exponential operations needed for the computation of the aforementioned illustrations. In the classical approach, the entire computation is separated into three phases. The $\ln {P}$ is computed using HV-CORDIC. Multiplication is performed to compute the $N{{\rm th}}$ power using linear rotation mode CORDIC (LR-CORDIC), and division is performed to compute the $N{{\rm th}}$ root using linear vectoring mode CORDIC (LV-CORDIC). In the last, the exponential is performed using the hyperbolic rotation mode CORDIC (HR-CORDIC). Figure 1 demonstrates this approach. If the HV-CORDIC is initialized with the inputs $Y^0=P-1$ and $X^0=P+1$ then the logarithm can be calculated as follows.

4 $$\begin{aligned} \tanh ^{-1}\left( \dfrac{P-1}{P+1} \right) = \dfrac{1}{2} \ln {P} \end{aligned}$$

From this discussion, it is clear that the outputs $X^N$ and $Y^N$ of the HV-CORDIC are not required for further calculation, and hence, the scale factor compensation is not required for HV-CORDIC. As shown in Fig. 1, the multiplication and division are performed for power and root computing using LR-CORDIC and LV CORDIC, respectively. HR-CORDIC computes the final exponential.

The problem with this architecture is that the values of P and N are limited by the convergence criteria of various classes of CORDIC algorithms. The range of P can be derived using the convergence criteria of HV-CORDIC, i.e. $\left| \tanh ^{-1}{\frac{Y^0}{X^0}}\right| \le 1.1182$ and input $X^0$ has to be positive. Based on the inputs ($Y^0=P-1$ and $X^0=P+1$ ) of HV-CORDIC, the range of P can be derived using the following constraints.

5 $$\begin{aligned} \left| \dfrac{P-1}{P+1} \right| \le tanh(1.1182) =0.807 \text { and } P+1>0 \end{aligned}$$

From the aforementioned constraints, the range of P can be worked out as follows:

6 $$\begin{aligned} P\in \left[ \dfrac{1}{9.36},9.36 \right] \end{aligned}$$

Such a small range of P limits the real-time applications of this standard architecture. From Eq. (5), it is clear that the input range of the HV class has to be increased to extend the range of P. For example, if HV-CORDIC can converge in the range, $\left| \tanh ^{-1}{\frac{Y^0}{X^0}}\right| \le 1.1182$, then the range of P can be extended to $P\in \left[ \dfrac{1}{403.43},403.43 \right]$. Two recent approaches have been proposed to expand the range of P. In the first approach, negative-indexed iterations were proposed for the HV and HR CORDICs. However, additional negative-indexed iterations increase the iterative stages, which require additional computational resources.

In the research^25^, authors have proposed to increase the convergence range by performing the negative-indexed iterations. The basic rotation angle of negative index iteration is $\left( 1-2^{2^{-j+1}}\right)$ as compared to $2^{-j}$ of standard CORDIC algorithm. The maximum rotation angle achieved by adding additional iterations is illustrated as follows:

7 $$\begin{aligned} \theta _{max} = \sum _{j=-m}^{0} \tanh ^{-1}\left( 1-2^{2^{-j+1}} \right) + \sum _{j=1}^{n} \tanh ^{-1}\left( 2^{-j} \right) \end{aligned}$$

The relation between m and the range of P is summarised in Table 2.

**Table 2:** Impact of *m* on range of *P*.

*m*	$\theta _{max}$	Range of* P*
0	2.099	$\left[ \dfrac{1}{66.67}, 66.67 \right]$
1	3.816	$\left[ \dfrac{1}{2067}, 2067\right]$
2	6.935	$\left[ \dfrac{1}{1.056\times 10^6}, 1.056\times 10^6 \right]$
3	12.827	$\left[ \dfrac{1}{1.384\times 10^11}, 1.384\times 10^11 \right]$

In the another research^26^, binary logarithms $(\log _2(\cdot ))$ and binary exponentials $(2^{(\cdot )})$ are used to compute the $N{{\rm th}}$ root and $N{{\rm th}}$ power, as illustrated in Eq. 8.

8 $$\begin{aligned} P^{\dfrac{1}{N}}&= 2^{\dfrac{\log _2{P}}{N}} \nonumber \\ P^N&=2^{N\log _2 P} \end{aligned}$$

The first step of this approach is to bring the range of P to the range that can be processed by BHV-CORDIC by means of the normalization of P. The normalization factor is always an integer power of two. As a result, this approach does not require performing additional negative index iterations. The value of *P* can be normalized as follows:

9 $$\begin{aligned} P=2^q \times p; \hspace{0.5cm}\text { where, } p\in [1,2] \end{aligned}$$

Later, the binary logarithm is calculated using a simple adder as follows:

10 $$\begin{aligned} \log _2 P = q + \log _2 p \end{aligned}$$

In the architecture presented in^26^, authors have used binary HV-CORDIC to compute $\log _2 p$. Similarly, the binary exponential $2^(\cdot )$ of the real number V is computed by decomposing the real number (V) into integer $(V_I)$ and fraction ($V_F$) parts as follows:

11 $$\begin{aligned} 2^V = 2^{V_I}\times 2^{V_F} \end{aligned}$$

In the above illustration, $V_I$ is the integer, and $2^{V_I}$ can be computed using left shift by $V_I$-bits. The $2^{V_F}$ is computed with a BHR-CORDIC. This method requires a small convergence range (i.e., $\vert Z_0 \vert \le 1$ ) of BHR-CORDIC as $V_F \in [0,1]$. As a result, this approach does not require performing the negative index iteration. However, both architectures suffer from very high hardware utilization, as radix-2 CORDIC generates one bit of precision in its one iteration. The selection criteria of R4HV-CORDIC to choose the amount of rotation is complicated. Also, the scale factor of R4HR-CORDIC is variable, and compensation necessitates the specific hardware. In this article, we have modified the architectures of R4HV and R4HR CORDICs to simplify the selection criteria and re-scaling of scale-factor for root and power computations. A proposed methodology brings down the complexity of radix-4 CORDIC below that of the standard algorithm.

## Proposed methodology

The high computation latency and hardware utilization of the existing design are the primary concerns, as radix-2 CORDIC produces 1-bit precision in each iteration. In the pipelined architecture, the insertion of parallelism between two iterations costs a lot of pipeline resources. The total computational latency of the architectures presented in^25^ and^26^ is 81 and 73, respectively. In the proposed design, we have attempted to reduce the latency and hardware utilization by introducing modified R4HV-CORDIC to compute the logarithm and R4HR-CORDIC to compute the exponential. The computational complexity of the high-radix CORDIC algorithm other than radix-4 is very high as all the selection functions are not the integer power of two. For example, the radix-8 CORDIC algorithm has a selection function ranging from -4 to +4, and the multiplication of the selection function with the coordinates requires four extra adders in each iteration. As a result, we have used the radix-4 CORDIC algorithm in the proposed design.

In the proposed methodology, the computation of $P^\frac{1}{N}$ and $P^N$ is based on the base-4 logarithm and the exponential, as given in the equations below.

12 $$\begin{aligned} P^{\dfrac{1}{N}}=4^{\left( \frac{\log _4{P}}{N}\right) } \nonumber \\ P^N=4^{\left( \log _4{P}*N\right) } \end{aligned}$$

We have used modified R4HV-CORDIC to compute $\log _4{\left( \cdot \right) }$ and R4HR-CORDIC to compute $4^{\left( \cdot \right) }$. The properties of natural hyperbolic rotation can also be proved for hyperbolic rotation in base-4 as given in^26^. For base-4 hyperbolic rotation, ${tanh}_4\left( a\right)$ can be defined as follows:

13 $$\begin{aligned} {tanh}_4\left( a\right) =\frac{4^a-4^{-a}}{4^a+4^{-a}} \end{aligned}$$

From the above illustration, the relation between the inverse hyperbolic function and the logarithm for base-4 can be computed as follows:

14 $$\begin{aligned} \tanh _4^{-1}\left( b\right) =0.5*\log _4{\frac{1+b}{1-b}} \end{aligned}$$

**Figure 2:**
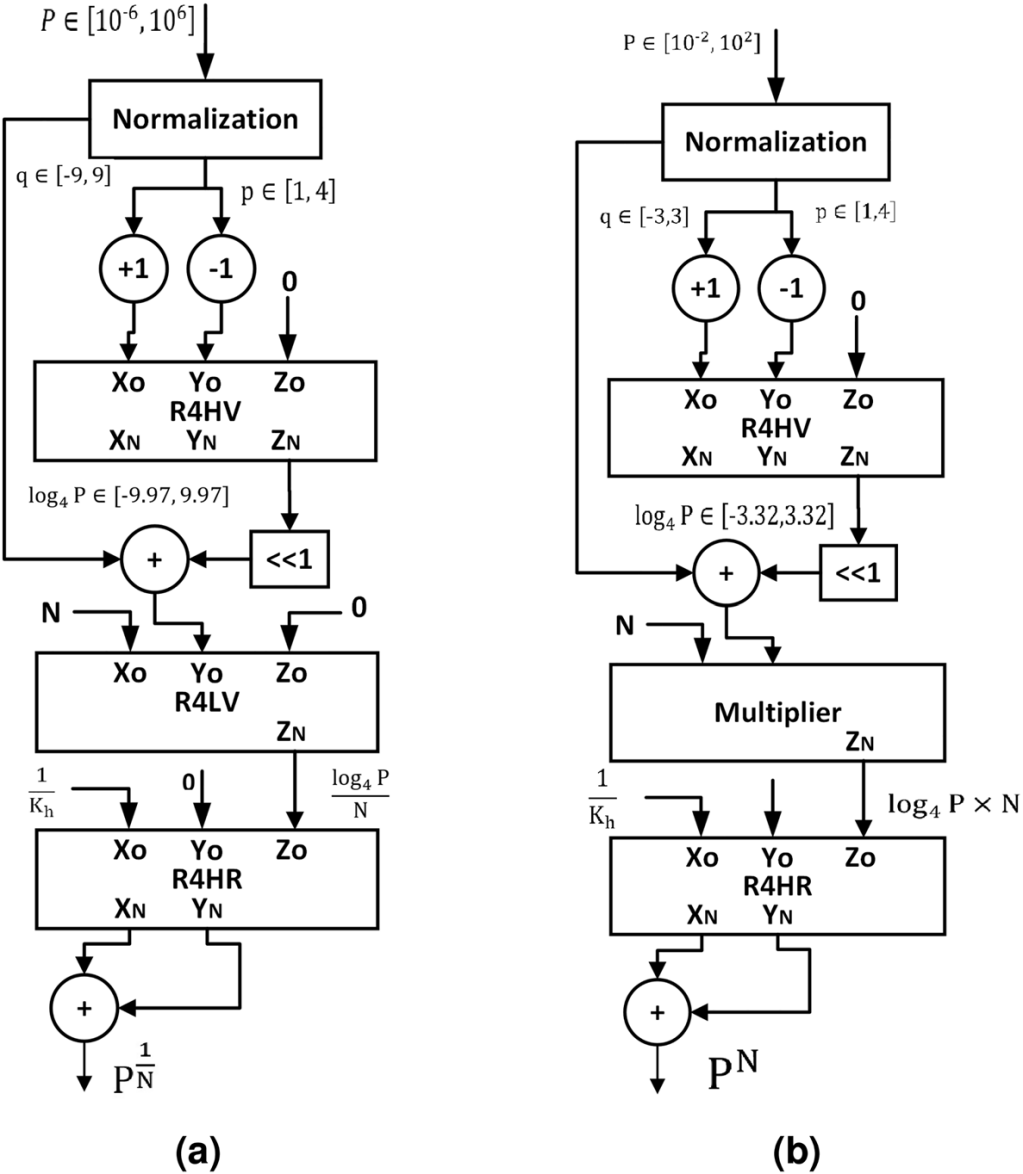
Proposed methodology to compute (a) $N{{\rm th}}$ root (b) $N{{\rm th}}$ power.

Figure 2a,b demonstrate the proposed root and power computation methodology, respectively. The range of variables at different stages is also shown in Fig. 2. The input range of the P is considered as $P\in \left[ 10^{-6}, 10^6 \right]$ and $P\in \left[ 10^{-2}, 10^2 \right]$ for root and power computation, respectively. The input range of R4HV-CORDIC is only $\left[ \dfrac{1}{4.19}, 4.19 \right]$, and it is discussed in the next section. Hence, the normalization is used to bring down the range of P to $p\in \left[ 1, 4 \right]$. The normalization is performed using relation $P=4^q \times p$; hence, the q is $\left[ -9, 9 \right]$ and $\left[ -3, 3 \right]$ after normalization for root and power computation, respectively. We have used the modified R4HV-CORDIC to compute the $\log _4{p}$, and $\log _4{P}$ can be computed by adding $\log _4{p}$ to *q* using the simple adder in both computations. In the next phase, R4LV-CORDIC is used to divide $\log _4{P}$ by *N* for root computation and a simple multiplier is used to multiply $\log _4{P}$ by *N* for power computation. Finally, the exponential required to compute $P^{\dfrac{1}{N}}$ and $P^{N}$ are calculated by R4HR CORDIC. The normalization is used to lower the input range of R4HR-CORDIC within the convergence range. The exponential $4^{(\cdot )}$ of the real number V is computed by decomposing the real number (V) into integer $(V_I)$ and fraction ($V_F$) parts as follows:

15 $$\begin{aligned} 4^V = 4^{V_I}\times 4^{V_F} \end{aligned}$$

In the proposed methodology, $4^{V_I}$ is computed using left shift by $2*V_I$ bits. The $4^{V_F}$ is computed with the R4HR-CORDIC. In the following section, the modified R4HV and R4HR CORDICs are discussed.

## Modified radix-4 CORDIC

The various classes of the radix-4 CORDIC algorithm that have been utilized in the proposed methodology are discussed here.

### Modified radix-4 HV-CORDIC

The R4HV-CORDIC can be defined as follows for base-4 logarithm computation.

16 $$\begin{aligned} \left[ \begin{array}{c} X^{j+1}\\ Y^{j+1}\\ \end{array}\right]&=\left[ \begin{array}{cc} 1&{}-\sigma _{j}4^{-j}\\ -\sigma _{j}4^{-j}&{}1\\ \end{array}\right] \left[ \begin{array}{c} X^{j} \\ Y^{j} \\ \end{array}\right] \nonumber \\ Z^{j+1}&=Z^{j}+{tanh}_4^{-1}\left( \sigma _j4^{-j}\right) \end{aligned}$$

where* j* is the integer starting with 1, and selection function $\sigma _j\ \in \left\{ -2,-1,0,1,2\right\}$. The radix-4 CORDIC does not require repeating any iteration for convergence. The aforementioned rotation introduces the scale factor which is given as $K=\prod _{j=1}^{\frac{n}{2}}\sqrt{\left( 1-\sigma _j^{-2}4^{-2j}\right) }$. The problem with the R4HV-CORDIC is the selection criteria to choose $\sigma _j$ and the complex scale factor K. Since we only use the value of the Z variable at the end of convergence, the re-scaling of the rotated vector is not required. However, in the R4HV-CORDIC algorithm, the selection criteria to choose the $\sigma _j$ are complex and depend on both coordinate values $X^j$ and $Y^j$. The convergence of the R4HV-CORDIC can be derived using the SRT-division method as given in^27,28^. According to the SRT division, the variable $Y^j$ is converted into a new variable as $W^j=4^jY^j$. After the conversion, the equation given in Eq. (16) will look as follows:

17 $$\begin{aligned} \left[ \begin{array}{c} X^{j+1}\\ W^{j+1}\\ \end{array}\right] =\left[ \begin{array}{cc} 1&{}-\sigma _j4^{-2j}\\ -{4\sigma }_j&{}4\\ \end{array}\right] \left[ \begin{array}{c} X^j\\ W^j\\ \end{array}\right] \end{aligned}$$

To guarantee the convergence of the algorithm, the variable $W^j$ must be bounded between the lower(L) and upper(U) limits which are defined as $L= \left( a-\frac{p}{r-1} \right) X^j$ and $U= \left( a+\frac{p}{r-1} \right) X^j$ for radix-r SRT division. According to the SRT divison method, to achieve maximum overlap between the intervals used for selecting different values of $\sigma _j$ and for minimal redundancy we have chosen $p=\dfrac{r}{2}$^27^. These limits for radix-4 SRT division can be defined as $L= \left( a-\frac{2}{3} \right) X^j$ and $U= \left( a+\frac{2}{3} \right) X^j$. We choose $\sigma _j=a$ according to the criteria given in equation Eq. (18) to guarantee convergence.

18 $$\begin{aligned} aX^j-\frac{2}{3}X^j\le W^j\le aX^j+\frac{2}{3}X^j \end{aligned}$$

The intervals to select the $\sigma _j$ can be derived using the criteria given in equation Eq. (18). The value of the variable $W^j$ should be bound within this interval in each iteration to ensure convergence. For example, to select $\sigma _j=2$, $W^j$ must fall within the interval $I_2:\left[ \frac{4}{3}X^j,\frac{8}{3}X^j\ \right]$. Similarly, to select $\sigma _j=1$, $W^j$ must fall within the interval $I_1:\left[ \frac{1}{3}X^j,\frac{5}{3}X^j\ \right]$. The overlapping between these two intervals is $\left[ \frac{4}{3}X^j,\frac{5}{3}X^j\ \right]$. Letter, we can select any value from this overlapping between two intervals. The criteria and overlapping intervals for a particular selection function are mentioned in Table 3.

**Table 3:** Overlapping intervals and criteria.

Selection function ($\sigma _j$)	Criteria	Overlapping intervals
2	$W^j\ge 1.5*X^j$	$\left[ \frac{4}{3}X^j,\frac{5}{3}X^j\ \right]$
1	$1.5*X^j\ge W^j\ge 0.5*X^j$	$\left[ \frac{1}{3}X^j,\frac{2}{3}X^j\ \right]$
0	${0.5*X^j\ge W}^j\ge -0.5*X^j$	$\left[ -\frac{2}{3}X^j,-\frac{1}{3}X^j\ \right]$
-1	${-0.5*X^j\ge W}^j\ge -1.5*X^j$	$\left[ -\frac{5}{3}X^j,-\frac{4}{3}X^j\ \right]$
-2	${-1.5*X^j\ge W}^j$	$\left[ -\frac{8}{3}X^j,-\frac{7}{3}X^j\ \right]$

**Table 4:** Selection criteria.

$\sigma _1$	$\sigma _0=2$			$\sigma _0=1$			$\sigma _0=0$			$\sigma _0=-1$			$\sigma _0=-2$		
	$Y_0,min$	$Y_0,max$	Selection criteria	$Y_0,min$	$Y_0,max$	Selection criteria	$Y_0,min$	$Y_0,max$	Selection criteria	$Y_0,min$	$Y_0,max$	Selection criteria	$Y_0,min$	$Y_0,max$	Selection criteria
2	2.546	2.698	2.625	0.970	1.054	1.012	0.182	0.233	0.207	− 0.291	− 0.261	− 0.275	− 0.606	− 0.589	− 0.59766
1	2.128	2.261	2.195313	0.738	0.812	0.773	0.043	0.087	0.063	− 0.375	− 0.348	− 0.359	− 0.653	− 0.638	− 0.64453
0	1.760	1.878	1.820313	0.533	0.599	0.566	− 0.080	− 0.041	− 0.063	− 0.448	− 0.425	− 0.438	− 0.693	− 0.680	− 0.6875
− 1	1.434	1.539	1.484375	0.352	0.410	0.383	− 0.189	− 0.154	− 0.172	− 0.513	− 0.492	− 0.500	− 0.730	− 0.718	− 0.72266
− 2	1.143	1.236	1.1875	0.191	0.242	0.219	− 0.286	− 0.255	− 0.270	− 0.571	− 0.553	− 0.563	− 0.762	− 0.752	− 0.75781

The convergence criteria for R4HV-CORDIC can be defined as follows:

19 $$\begin{aligned} \theta _{max}=\sum _{j=1}^{\frac{n}{2}}\tanh _4^{-1}{\left( {\sigma _{j,max}4}^{-j}\right) }=0.5169 \end{aligned}$$

The inputs to R4HV-CORDIC are $Y_0=p-1$ and $X_0=p+1$. The range of p is limited by the convergence range discussed in Eq. (5), and it can be derived using the constraints given below.

20 $$\begin{aligned} \left| \frac{p-1}{p+1}\right| \le \tanh _4{\left( 0.5169\right) }=0.6148 \text { and } p+1>0 \end{aligned}$$

From the above constraints, the range of p can be derived as $p\in \left[ \frac{1}{4.19},4.19\right]$. As discussed earlier, this small convergence range is enough as the output of the normalizer is between 1 and 4 for the proposed architecture.

The problem with the R4HV-CORDIC is that the $\sigma _j$ depends on both the coordinates $X^j$ and $W^j$. The computation of the selection function is very complex, as in each iteration $W^j$ needs to be compared with the complex selection criteria given in Table 3. The computation of $0.5*X^j$ can be achieved with a simple binary shift, and additional hardware is not required. However, to compute $1.5*X^j$, an additional adder may be required. In this section, we have discussed the methodology to simplify the selection criteria for the application of the root and power computations.

Since the inputs to radix-4 HV CORDIC are fixed ($Y_0=p-1$ and $X_0=p+1$), we can derive the selection criteria to choose $\sigma _j$, which only depends on the variable $Y_j$ for any iteration index j. Because of the fixed inputs to R4HV-CORDIC, the variables $Y_j$ and $X_j$ can also be represented in terms of $Y_0$ for any iteration index, j. For example, $Y_1$ and $X_1$ can be represented in terms of $Y_0$ using the identities $X_0=Y_0+2$ and Eq. (16) as follows:

21 $$\begin{aligned} Y_1&=Y_0 \left( 1-\frac{\sigma _0}{4} \right) -\frac{\sigma _0}{2} \nonumber \\ X_1&=Y_0 \left( 1-\frac{\sigma _0}{4} \right) +2 \end{aligned}$$

From the identities given in Eq. (21), the relation between $Y_1$ and $X_1$ can be derived as follows:

22 $$\begin{aligned} X_1=Y_1+2+\frac{\sigma _0}{2} \end{aligned}$$

Now criteria to select $\sigma _0$ and $\sigma _1$ can be derived using the overlapping intervals given in Table 3 and the identities given in Eq. (22) as folloes:

23 $$\begin{aligned} aX_1\le 16Y_1 \le bX_1 \end{aligned}$$

Where $[aX_1,bX_1 ]$ is the overlapping interval shown in Table 3 and $a\in \lbrace 4/3,1/3,-2/3,-5/3,-8/3 \rbrace$, and $b\in \lbrace 5/3,2/3,-1/3,-4/3,-7/3 \rbrace$. Using the identity $X_1=Y_1+2+\frac{\sigma _0}{2}$ and generalized criteria given in Eq. (23), we can derive an interval to select a particular selection function as follows:

24 $$\begin{aligned} \frac{4a}{16-a} \le Y_1 \le \frac{4b}{16-b} \end{aligned}$$

Now, using Eq. (21), $Y_1$ can be represented in terms of $Y_0$ and the selection criteria given in 24 can be rewritten as follows:

25 $$\begin{aligned} \frac{\frac{4a}{16-a}+\frac{\sigma _0}{2}}{1-\frac{\sigma _0}{4}} \le Y_0 \le \frac{\frac{4b}{16-b}+\frac{\sigma _0}{2}}{1-\frac{\sigma _0}{4}} \end{aligned}$$

The range of $Y_0$ mentioned in the Eq. (25) can be used to select $\sigma _0$ and $\sigma _1$. By choosing the appropriate value of $\sigma _0$, a, and b, we can derive the selection criteria to select $\sigma _1$. For example, if we choose $\sigma _0=2$, $a=\frac{4}{3}$, and $b=\frac{5}{3}$ then the range of $Y_0$ to select $\sigma _0=2$ and $\sigma _1=2$ is found out as $2.54 \le Y_0 \le 2.69$.

**Table 5:** Selection criteria to select $\sigma _{2}$.

$\sigma _{0}$	$\sigma _{1}$	Range of $64Y_2$ to select $\sigma _2$										Comparison points				
		$\sigma _2=2$		$\sigma _2=1$		$\sigma _2=0$		$\sigma _2=-1$		$\sigma _2=-2$		$A_{2}$	$A_{1}$	$A_{0}$	$A_{-1}$	$A_{-2}$
2	2	9.19	11.55	2.27	4.54	− 4.45	− 2.24	− 10.97	− 8.82	− 17.28	− 15.19	10.5	3.5	− 3.5	− 10	−16
2	1	8.68	10.91	2.14	4.30	− 4.21	−2.11	− 10.36	−8.33	− 16.32	−14.35	9.75	3.25	− 3.25	−9.5	− 15.25
2	0	8.17	10.27	2.01	4.04	−3.96	− 1.98	−9.74	− 7.83	−15.36	− 13.50	9.25	3	−3	− 8.75	−14.5
2	− 1	7.65	9.63	1.88	3.79	−3.71	− 1.87	−9.14	− 7.35	−14.40	− 12.66	8.75	2.75	−2.75	− 8.25	−15.5
2	− 2	7.16	8.99	1.75	3.53	−3.47	− 1.74	−8.52	− 6.86	−13.44	− 11.81	8	2.5	−2.5	− 7.75	−12.75
1	2	7.65	9.63	1.88	3.79	− 3.71	−1.87	− 9.14	−7.35	− 14.40	−12.66	8.625	2.875	− 2.75	−8.25	− 13.5
1	1	7.23	9.09	1.78	3.58	−3.51	− 1.77	−8.63	− 6.94	−13.59	− 11.96	8.125	2.625	−2.625	− 7.75	−12.75
1	0	6.81	8.55	1.68	3.37	− 3.30	−1.66	− 8.13	−6.53	− 12.80	− 11.25	7.625	2.5	− 2.5	− 7.375	− − 12
1	− 1	6.39	8.03	1.57	3.16	− 3.10	− 1.55	− 7.62	− 6.12	− 11.99	− 10.55	7.25	2.375	− 2.375	− 6.875	− 11.25
1	− 2	5.95	7.49	1.47	2.94	− 2.89	− 1.45	− 7.10	− 5.71	− 11.20	− 9.84	6.75	2.25	− 2.125	− 6.375	− 10.5
0	2	6.13	7.71	1.51	3.03	− 2.97	− 1.50	− 7.31	− 5.88	− 11.52	− 10.12	7	2.75	− 2.25	− 6.5	− 10.875
0	1	5.79	7.27	1.42	2.87	− 2.80	− 1.41	− 6.90	− 5.56	− 10.88	− 9.56	6.5	2.125	− 2.125	− 6.25	− 10.25
0	0	5.45	6.85	1.34	2.70	− 2.64	− 1.33	− 6.50	− 5.22	− 10.24	− 9.01	6.125	2	− 2	− 5.875	− 9.625
0	− 1	5.11	6.41	1.25	2.52	− 2.47	− 1.24	− 6.09	− 4.90	− 9.60	− 8.45	5.75	1.875	− 1.875	− 5.5	− 9
0	− 2	4.76	5.99	1.18	2.36	− 2.30	− 1.16	− 5.68	− 4.57	− 8.96	− 7.88	5.375	1.75	− 1.75	− 5.125	− 8.375
− 1	2	4.60	5.77	1.13	2.28	− 2.23	− 1.11	− 5.48	− 4.40	− 8.64	− 7.60	5.125	1.75	− 1.75	− 5	− 8.125
− 1	1	4.34	5.45	1.06	2.15	− 2.10	− 1.06	− 5.18	− 4.16	− 8.15	− 7.18	4.875	1.625	− 1.625	− 4.625	− 7.625
− 1	0	4.08	5.13	1.01	2.02	− 1.98	− 1.00	− 4.88	− 3.92	− 7.68	− 6.76	4.625	1.5	− 1.5	− 4.375	− 7.25
− 1	− 1	3.83	4.81	0.95	1.89	− 1.86	− 0.93	− 4.57	− 3.67	− 7.19	− 6.34	4.375	1.5	− 1.375	− 4.125	− 6.75
− 1	− 2	3.57	4.49	0.88	1.77	− 1.73	− 0.87	− 4.26	− 3.43	− 6.72	− 5.91	4	1.375	− 1.25	− 3.875	− 6.375
− 2	2	3.06	3.85	0.76	1.51	− 1.48	− 0.74	− 3.66	− 2.94	− 5.76	− 5.07	3.5	1.125	− 1.125	− 3.375	− 5.375
− 2	1	2.89	3.64	0.72	1.43	− 1.41	− 0.70	− 3.46	− 2.78	− 5.44	− 4.79	3.25	1	− 1	− 3.125	− 5.125
− 2	0	2.73	3.42	0.67	1.34	− 1.32	− 0.67	− 1.24	− 0.63	− 1.15	− 0.58	3	1	− 1	− 2.875	− 4.875
− 2	− 1	2.55	3.21	0.63	1.27	− 1.24	− 0.63	− 3.05	− 2.44	− 4.80	− 4.22	2.875	1	− 1	− 2.75	− 4.5
− 2	− 2	2.38	3.00	0.59	1.18	− 1.15	− 0.58	− 2.84	− 2.29	− 4.48	− 3.94	2.625	0.875	− 0.875	− 2.5	− 4.25

Similarly, the range of $Y_0$ can be found for all possible combinations of $\sigma _0$ and $\sigma _1$ by iterating the equation Eq. (25). The range of $Y_0$ to select various values of $\sigma _0$ and $\sigma _1$ is summarized in Table 4, and from this range, criteria to select the selection function is shown in the adjutant column in Table 4. All the values of the criteria can be represented using 10-bit, and as a result, a 10-bit comparator is required for the comparison. The problem with this method is that the number of comparison points increases exponentially to the iteration index. For example, the variable $Y_0$ has to be compared with 125 selection criteria to select $\sigma _0$, $\sigma _1$, and $\sigma _2$. In the proposed architecture, values for $\sigma _0$ and $\sigma _1$ are selected by comparing the value of $Y_0$ with the criteria given in Table 4. The proposed method to select the $\sigma _j$ for the iteration index $j\ge 2$ is discussed next.

As given in^29^, the criteria to select $\sigma _2$ can be used to select $\sigma _j$ for all the iterations with iteration index $j\ge 2$. In the proposed architecture, criteria to select $\sigma _2$ are stored on a look-up table, and they are used to decide the value of $\sigma _j$ for the rest of the iterations. As discussed earlier, the relation between variables $X_2$ and $Y_2$ can be derived using the identity $X_1=Y_1+2+\frac{\sigma _0}{2}$ and iteration equation for $j=2$ as follows:

26 $$\begin{aligned} X_2=Y_2+2\left( 1+\frac{\sigma _0}{4} \right) \left( 1+\frac{\sigma _1}{16} \right) \end{aligned}$$

Now, the range of $Y_2$ needed to select $\sigma _2$ can be derived using the identity $aX_2 \le 64Y_2 \le bX_2$ as follows:

27 $$\begin{aligned} \left( \frac{2a}{64-a} \right) \left( 1+\frac{\sigma _0}{4} \right) \left( 1+\frac{\sigma _1}{16} \right) \le Y_2 \le \left( \frac{2b}{64-b} \right) \left( 1+\frac{\sigma _0}{4} \right) \left( 1+\frac{\sigma _1}{16} \right) \end{aligned}$$

Now the above equation can be iterated with various values of $\sigma _{0}$ and $\sigma _{1}$ to find out the five criteria points $A_{i}$ for $\sigma _j=i$. The range of $Y_2$ to select $\sigma _j$ for various values of $\sigma _{0}$ and $\sigma _{1}$ is summarised in Table 5. The last five columns of Table 5 show the criteria to select $\sigma _j$ for various values of $\sigma _{0}$ and $\sigma _{1}$. All the values of the comparison points can be represented using 8-bit, which results in only an 8-bit comparator. Since each comparison point can be represented using 8-bit, a look-up table with a size of $125\times 8$ bits is required to store all the criteria. Once the values of $\sigma _0$ and $\sigma _1$ are known, the comparison points from the look-up table can be loaded into registers. Later, these comparison points will be used to select the value of $\sigma _j$ for the rest of the iterations.

The computation flow of the proposed modified R4HV-CORDIC is presented in Table 6. Table 6 states the computation performed by the* X*,* Y*, and* Z* data paths in each iteration. In the first step, a normalization procedure is performed by evaluating the values of q and p using the identity $4^q \le P \le 4^{q+1}$ where q is an integer number. By performing a normalization process, P is converted to the convergence range of R4HV-CORDIC as $p\in \left[ 1, 4 \right]$, and the logarithm of P is computed as $\log _4{P}=q+\log _4{p}$. Later, the $X_{0}$ and $Y_{0}$ are initialised as $X_{0}=p+1$ and $Y_{0}=p-1$. The *Z*-datapath computes $\sigma _0$ and $\sigma _1$ by comparing $Y_{0}$ with the selection criteria given in Table 4. Since $\sigma _0$ and $\sigma _1$ are already known, the next two stages compute the R4HV-CORDIC iterations with indexes j = 1, 2. Stage 2 also loads the values of comparison points from the look-up table based on the values of $\sigma _0$ and $\sigma _1$. These comparison points will be used to decide the value of $\sigma _j$ for the following iterations. In the third stage of the proposed algorithm, first, *Z*-datapath evaluates the value of $\sigma _j$ by comparing the $Y_2$ with the comparison points retrieved from the look-up table in the previous stage. The rest of the stages follow this process to get convergence. The architecture of the proposed algorithm is discussed next.

**Table 6:** Computational flow of the modified R4HV-CORDIC.

Iteration index (j) / Stage	Datapath			Operations to be performed
	*X*	*Y*	*Z*	
$j=0$, Pre-processing	$X_0=p+1$	$Y_0=p-1$	Evaluate: $\sigma _0$, $\sigma _1$	Compute $X_0$ and $Y_0$. Evaluate $\sigma _0$ and $\sigma _1$ by comparing p with the criteria given in Table 4.
$j=1$	$X_1=X_0-\frac{\sigma _0Y_0}{4}$	$Y_1=Y_0-\frac{\sigma _0X_0}{4}$	$Z_1=Z_0+{tanh}_4^{-1}\left( \frac{\sigma _0}{4}\right)$	Compute $X_1$, $Y_1$, and $Z_1$.
$j=2$	$X_2=X_1-\frac{\sigma _1Y_1}{16}$	$Y_2=Y_1-\frac{\sigma _1X_1}{16}$	$Z_2=Z_1+{tanh}_4^{-1}\left( \frac{\sigma _1}{16}\right)$	Compute $X_2$, $Y_2$, and $Z_2$. Load comparison points $A_{i}$ from the look-up table.
$j=3$ to $\frac{n}{2}$	$X_j=X_{j-1}-\frac{\sigma _{j-1}Y_{j-1}}{4^{j-1}}$	$Y_j=Y_{j-1}-\frac{\sigma _{j-1}X_{j-1}}{4^{j-1}}$	$Z_j=Z_{j-1}+{tanh}_4^{-1}\left( \frac{\sigma _{j-1}}{4^{j-1}}\right)$	Evaluate $\sigma _{j}$ by comparing $Y_{j}$ with the comparison points loaded from the look-up table in the second iteration, compute $X_j$, $Y_j$, and $Z_j$.

#### The modified R4HV-CORDIC architecture to compute $\log _4{P}$

The process of calculating $\log _4{P}$ is divided into three stages. The initial stage is pre-processing, where the range of P is transformed to the input range of R4HV-CORDIC. The second stage employs the proposed modified R4HV-CORDIC to calculate $\log _4{p}$. Finally, the post-processing stage computes $\log _4{P}$ by adding q to $\log {p}+q$. The following section discusses each step in detail.

**Figure 3:**
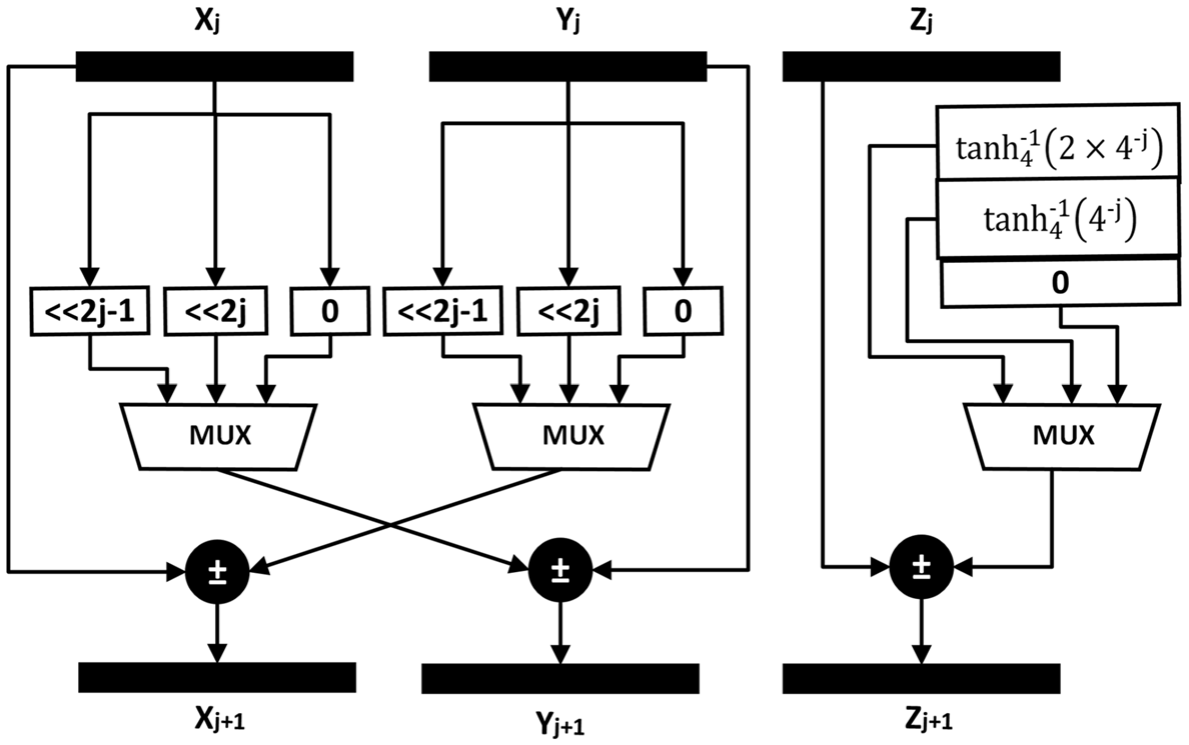
Architecture of R4HV-CORDIC stage.

The input range of the R4HV-CORDIC is $p\in \left[ \frac{1}{4.19}, 4.19 \right]$. In the pre-processing stage, the value of *P* is normalized with factor q in such a way that normalized p is in the range $p\in \left[ 1, 4 \right]$. The normalization can be achieved by right-shifting P by 2q-bits for $4^q \le P \le 4^{q+1}$, and q can be found out using the simple combinational logic. The relation between the actual value of P and normalized p can be expressed as $P=p \times 4^q$. In addition to normalizing p, the pre-processing stage calculates the value of $X_0$ and $Y_0$ by adding and subtracting normalized p with 1. The pre-processing stage also involves comparing the normalized p with the conditions specified in Table 4 to determine the values of $\sigma _0$ and $\sigma _1$. This comparison necessitates a 10-bit comparator, as discussed earlier. The normalization can be achieved by binary shift using a fixed number of bits in fixed-point representation and its delay can be ignored. The radix-4 HV CORDIC receives $X_0$, $Y_0$, $\sigma _0$, and $\sigma _1$ from the pre-processing stage and computes the $\log _4{\left( p\right) }$. The generalized architecture of *X*-*Y* datapaths of the R4HV-CORDIC is shown in Fig. 3. The adder/subtractor and shifter are the basic components of the* X* and* Y* datapaths, and the architecture of the* X* and* Y* data paths is the same for all stages except for the shift value. The shifter can be implemented using a simple 3-to-1 multiplexer and it multiplies the $\sigma _j$ with $X_j$ (in Y-datapath) and $Y_j$ (in* X*-datapath). The multiplication can be achieved using binary shift as the value of $\sigma _j$ is always an integer power of two. The *Z*-datapath of the first stage first access the $\tanh _4^{-1}\left( \frac{\sigma _0}{4}\right)$ from the ROM table and add or subtract it to $Z_0$ to generate the $Z_1$. Since there are three values of $sigma_j$ and negative values of rotation angle can be added by performing subtraction, only three values of $\tanh _4^{-1}\left( \frac{\sigma _0}{4}\right)$ need to be pre-computed. The *Z*-datapath of the second stage pre-loads the comparison points to pipelined registers from the ROM table based on the value of $\sigma _0$ and $\sigma _1$. The *Z*-datapath of the third stage compares the comparison points ($A_i$) received from the previous computation with the $64Y_2$ to derive $\sigma _2$. Since the X and Y datapaths can only compute after the computation of $\sigma _2$, the critical path delay of the *X*-*Y* datapath has an additional delay of a comparator, as compared to the first and second stages. In the proposed architecture, we have proposed pipelined structure where each stage is separated with pipeline registers so that they can compute in parallel. Table 7 summarizes the critical path delay of the *X*-*Y* and *Z* rotators of each stage.

**Table 7:** Critical path delay of R4HV-CORDIC.

Stage	*X*-*Y* Datapath	*Z* Datapath
Pre-processing	$T_{add}$	$T_{add}+T_{comp}$
First and Second stages of radix-4 HVCORDIC	$T_{mux}+T_{add}$	$T_{ROM}+T_{add}$
Rest of the stages of radix-4 HVCORDIC	$T_{comp}+T_{add}+T_{mux}$	$T_{comp}+T_{add}+T_{ROM}$
Post-processing		$T_{add}$

$T_{NORM}$: Delay of Normalizer; $T_{add}$: Delay of adder subtractor; $T_{comp}$: Delay of comparator; $T_{ROM}$: Delay time to access ROM table

The critical path delays of all rotators are approximately the same as they use an adder/subtractor and comparator, as shown in Table 7. Radix-4 CORDIC rotation requires half the iterations of radix-2 for the same N-bit precision^29^. The proposed modified R4-HV CORDIC algorithm introduces only a minimal overhead of three 3-to-1 multiplexers in each stage. Therefore, implementing the modified R4-HV CORDIC algorithm requires $\dfrac{3N}{2}$ adders and $\dfrac{3N}{2}$ 3-to-1 multiplexers, which is significantly less than the 3N adders required by the radix-2 CORDIC algorithm. The proposed R4-HV CORDIC algorithm has better hardware utilization than the radix-2 CORDIC algorithm since the complexity of an adder is approximately twice that of a 3-to-2 multiplexer^30^. In the post-processing stage, the output of the *Z*-datapath of the last stage of the radix-4 HVCORDIC is shifted right by 1-bit to generate $\log _4{p}$. Later, the adder adds $\log _4{p}$ with the normalized shift value q in the post-processing stage to generate $\log _4{P}$, which has a delay of one adder.

### Radix-4 LV-CORDIC

The radix-4 LV-CORDIC has the most straightforward architecture among all the versions of CORDIC. The computational equations of R4LV-CORDIC are given in Eq. (28).

28 $$\begin{aligned} \left[ \begin{array}{c} X^{j+1}\\ Y^{j+1}\\ \end{array}\right]&=\left[ \begin{array}{cc} 1&{}-\sigma _{j}4^{-j}\\ 0 &{}1\\ \end{array}\right] \left[ \begin{array}{c} X^{j} \\ Y^{j} \\ \end{array}\right] \nonumber \\ Z^{j+1}&=Z^{j}+\sigma _j4^{-j} \end{aligned}$$

**Figure 4:**
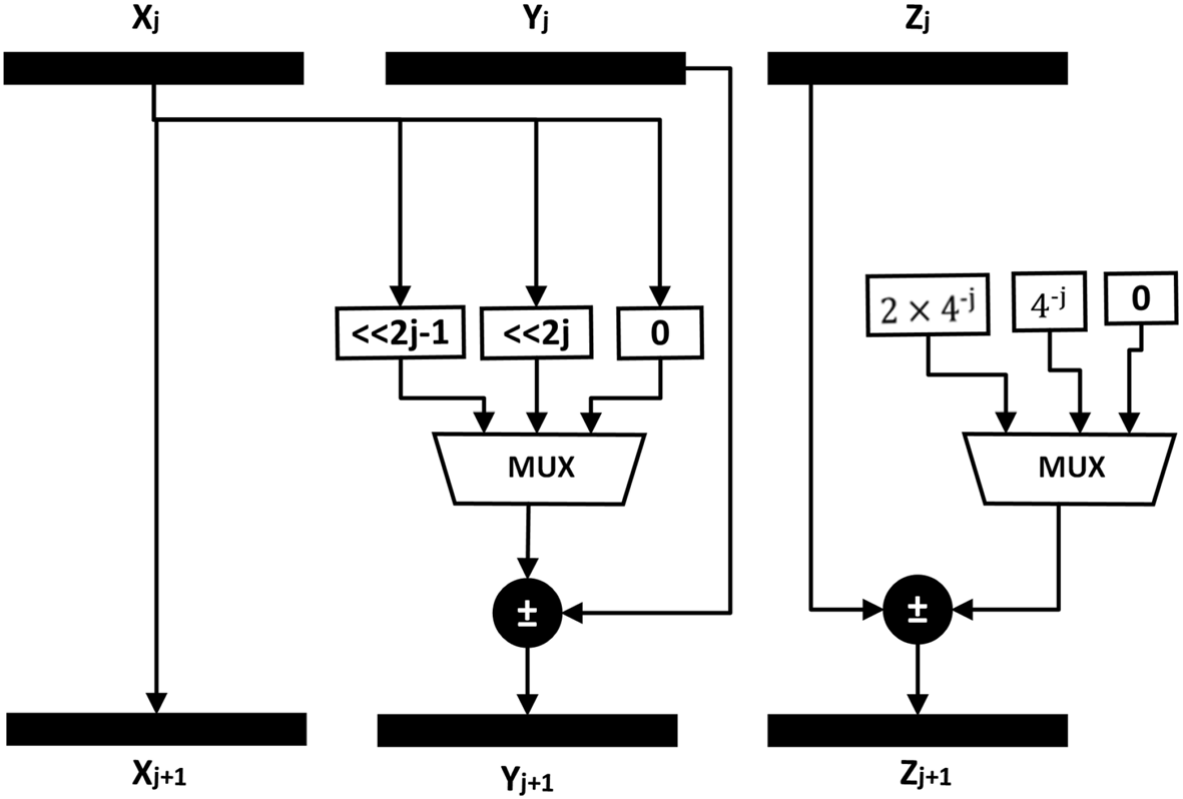
Architecture of R4LV-CORDIC.

For R4LV-CORDIC, iteration index j starts from 0. The extension of the input range of the R4LV-CORDIC can be increased by performing the non-positive index iteration with the same architecture, and additional hardware is not required. The implementation of R4LV-CORDIC necessitates the two multiplexers and adders, each for Y and Z datapaths, as depicted in Fig. 4. The critical path delay of Y and Z rotators includes the delay of one adder and multiplexer. As compared to the radix-2 CORDIC algorithm that uses two adders, one stage of R4-LV CORDIC uses two adders and two 3-to-1 multiplexers. However, the R4-LV CORDIC algorithm achieves convergence in half the iteration. The total hardware complexity of R4-LV CORDIC is N adders and N multiplexers, which is less than the 2N adders used by the radix-2 algorithm. Also, LV mode of the CORDIC does not generate scaling and compensation of the scale factor is not required in LV class of the CORDIC.

### Modified radix-4 HR-CORDIC

In this section, the radix-4 hyperbolic rotation CORDIC is discussed. The R4HR-CORDIC is used to determine the exponential $(4^{(\cdot )})$ in the proposed method. The R4HR-CORDIC iteration can be illustrated as follows:

29 $$\begin{aligned} \begin{bmatrix} X^{j+1} \\ Y^{j+1} \end{bmatrix}&= \begin{bmatrix} 1 &{} \sigma ^{j}4^{-j} \\ \sigma ^{j}4^{-j} &{} 1 \end{bmatrix} \begin{bmatrix} X^{j} \\ Y^{j} \end{bmatrix} \nonumber \\ Z^{j+1}&= Z^j - \tanh _{4}^{-1}\left( \sigma ^{j}4^{-j}\right) \end{aligned}$$

where $\sigma ^{j} \in \lbrace -2, -1, 0, 1, 2 \rbrace$, and $j=1, 2, ...,\frac{n}{2}$. The ($X_j$, $Y_j$) is the input vector, and ($X_{j+1}$, $Y_{i+1}$) represents the output vector after $j{{\rm th}}$ rotation. After the convergence, the final coordinates $X_n$ and $Y_n$ of the rotated vector are as follows:

30 $$\begin{aligned} X_n=K_h \cosh _4 \left( Z_1 \right) \nonumber \\ Y_n=K_h \sinh _4 \left( Z_1 \right) \end{aligned}$$

Where $K_h$ is the scale factor. From above equation 30, exponential $4^{Z_1}$ can be computed as follows:

31 $$\begin{aligned} 4^{Z_1}=K_h\left( \cosh _4 \left( Z_1 \right) +\sinh _4 \left( Z_1 \right) \right) \end{aligned}$$

However, the computation of the final exponential requires the scale-factor compensation. The scale factor is given by $K_h = \prod _{j=1}^{\frac{n}{2}} \left( \sqrt{1-\sigma _j^2 4^{-2j}} \right)$. The variable scale factor is the disadvantage of the R4HR-CORDIC. Another problem with R4HR-CORDIC is the convergence range. According to the illustration given in eq1, the minimum convergence range required is $\left| Z_1 \right| \le 1$. However, the convergence range of radix-4 HR CORDIC is only $\left| Z_1 \right| \le 0.501$. In the proposed architecture, an attempt is made to address these issues. In the next section, the convergence of the proposed CORDIC algorithm, its range of convergence, and scale factor compensation are discussed.

#### Proposed modified Radix-4 HR CORDIC

The high-radix CORDIC algorithm helps achieve convergence faster than the standard CORDIC algorithm. However, the scale factor of the high-radix CORDIC algorithm is complex, and its compensation may require significant hardware. In the proposed architecture, we have used the Taylor series approximation of hyperbolic sine and cosine for the high-radix CORDIC algorithm to achieve the scaling-free rotation. The Taylor series approximation of $\sinh (\theta )$ and $\cosh (\theta )$ with angle $\theta =\sigma _j4^{-j}$ can be defined as follows:

32 $$\begin{aligned} \sinh \left( \sigma _j4^{-j} \right)&=\sigma _j4^{-j}+\frac{\sigma _j^34^{-3j}}{3!}+\frac{\sigma _j^54^{-5j}}{5!}+\ldots \nonumber \\ \cosh \left( \sigma _j4^{-j} \right)&=1+\frac{\sigma _j^24^{-2j}}{2!}+\frac{\sigma _j^44^{-4j}}{4!}+\ldots \end{aligned}$$

The computation of a high-order Taylor approximation requires substantial hardware, and it is advisable to use a low-complexity Taylor approximation. The Taylor estimation is therefore constrained to two terms in the proposed design. The effect of iteration on accuracy in terms of binary bits (n) has to be studied for the potential error in the representation of the rotation vector. From Eq. (32), the term $\frac{\sigma _j^54^{-5j}}{5!}$ of the Taylor approximation of sinh can be ignored if $\frac{\sigma _j^54^{-5j}}{5!} \le 2^{-n}$. From this relation, we can conclude that the term $\frac{\sigma _j^54^{-5j}}{5!} \le 2^{-n}$ can be ignored for iteration index $j\ge \frac{n-2}{10}$. Similarly, term $\frac{\sigma _j^44^{-4j}}{4!}$ can be ignored for the iteration index $j\ge \frac{n-1}{8}$. For example, if 32-bit precision is targeted, then the terms $\frac{\sigma _j^54^{-5j}}{5!}$ and $\frac{\sigma _j^44^{-4j}}{4!}$ can be ignored for iteration index $j\ge 3$ and $j\ge 4$, respectively, without introducing any quantization error. The effective word length ($WL_E$) is another measure to check the error performance in the two-dimensional rotation. As given in^31,32^, the $WL_E$ for two-dimensional rotation can be defined as follows:

33 $$\begin{aligned} WL_E= -\log _2 \epsilon + 1.5 \end{aligned}$$

where, $\epsilon = \sqrt{\epsilon _C^2 + \epsilon _S^2}$, and $\epsilon _C$ and $\epsilon _S$ es are the absolute errors generated by the Taylor approximation in the cosine and sine components, respectively.

**Table 8:** ROM table.

$256 Y_0$	$\sigma _0$	$\sigma _1$	$\sigma _2$	$\sigma _3$	$K_{h}$	$256Y_0$	$\sigma _0$	$\sigma _1$	$\sigma _2$	$\sigma _3$	$K_{h}$	$256Y_0$	$\sigma _0$	$\sigma _1$	$\sigma _2$	$\sigma _3$	$K_{h}$
253	1	2	1	2	0.674	167	1	1	− 1	− 1	0.754	83	0	2	− 2	2	0.859
250	1	2	1	1	0.675	164	1	1	− 2	2	0.750	80	0	2	− 2	1	0.859
247	1	2	1	0	0.675	161	1	1	− 2	1	0.750	77	0	2	− 2	0	0.859
244	1	2	1	− 1	0.675	158	1	1	− 2	0	0.750	75	0	1	2	2	0.960
241	1	2	0	2	0.676	155	1	1	− 2	− 1	0.750	72	0	1	2	1	0.961
238	1	2	0	1	0.676	152	1	1	− 2	− 2	0.750	69	0	1	2	0	0.961
235	1	2	0	0	0.676	151	1	0	1	2	0.779	66	0	1	2	− 1	0.961
232	1	2	0	− 1	0.676	148	1	0	1	1	0.779	63	0	1	1	2	0.966
230	1	2	− 1	2	0.674	145	1	0	1	0	0.779	60	0	1	1	1	0.966
227	1	2	− 1	1	0.675	143	1	0	1	− 1	0.779	57	0	1	1	0	0.966
224	1	2	− 1	0	0.675	140	1	0	0	2	0.780	54	0	1	1	− 1	0.966
221	1	2	− 1	− 1	0.675	137	1	0	0	1	0.781	51	0	1	0	2	0.968
218	1	2	− 2	2	0.670	134	1	0	0	0	0.781	49	0	1	0	1	0.968
215	1	2	− 2	1	0.671	131	1	0	0	− 1	0.781	46	0	1	0	0	0.968
212	1	2	− 2	0	0.671	129	0	2	2	2	0.859	43	0	1	0	− 1	0.968
210	1	1	2	2	0.750	126	0	2	2	1	0.859	40	0	1	− 1	2	0.966
207	1	1	2	1	0.750	123	0	2	2	0	0.859	37	0	1	− 1	1	0.966
204	1	1	2	0	0.750	120	0	2	2	− 1	0.859	34	0	1	− 1	0	0.966
201	1	1	2	− 1	0.750	117	0	2	1	2	0.864	31	0	1	− 1	− 1	0.966
198	1	1	1	2	0.754	114	0	2	1	1	0.864	28	0	1	− 2	2	0.960
196	1	1	1	1	0.754	112	0	2	1	0	0.864	25	0	1	− 2	1	0.961
193	1	1	1	0	0.754	109	0	2	1	− 1	0.864	23	0	1	− 2	0	0.961
190	1	1	1	− 1	0.754	106	0	2	0	2	0.866	22	0	0	2	0	0.992
187	1	1	0	2	0.756	103	0	2	0	1	0.866	19	0	0	2	− 1	0.992
184	1	1	0	1	0.756	100	0	2	0	0	0.866	16	0	0	1	2	0.998
181	1	1	0	0	0.756	97	0	2	0	− 1	0.866	13	0	0	1	1	0.998
178	1	1	0	− 1	0.756	94	0	2	− 1	2	0.864	10	0	0	1	0	0.998
175	1	1	− 1	2	0.754	91	0	2	− 1	1	0.864	7	0	0	1	− 1	0.998
172	1	1	− 1	1	0.754	88	0	2	− 1	0	0.864	4	0	0	0	2	0.999
170	1	1	− 1	0	0.754	85	0	2	− 1	− 1	0.864	1	0	0	0	1	1.000

The $WL_E$ of the hyperbolic rotation for the proposed Taylor approximation for iteration index $j = 4$ is 38 bits. It indicates that this rotation may generate an error in the $38{{\rm th}}$ bit. The Taylor approximation is more accurate for the smaller values of the rotation angle, and as a result, the $WL_E$ will be improved for higher iteration indices. The two terms of the Taylor approximation of hyperbolic sine and cosine are used for iteration index $j \ge 4$. The terms $\frac{\sigma _j^34^{-3j}}{3!}$ from a sine approximation and $\frac{\sigma _j^24^{-2j}}{2!}$ from a cosine approximation can be ignored without any error for iteration index $j\ge 6$ and $j\ge 12$, respectively. This way, the hardware required to compute the scaling-free rotation is reduced for higher values of j. However, the scale factor generated by the first three iterations needs to be compensated. The scale factor of the high-radix CORDIC algorithm depends on $\sigma _j$. In the proposed algorithm, the $\sigma _j$ of the first three iterations is pre-computed by comparing the rotation angle $(Z_0)$ with the selection criteria. Once the value of $\sigma _j$ is known, the scale factor can be pre-computed and stored on a ROM table. Initializing the coordinate values with the pre-computed scale factor can result in compensation of the scale factor. This method is discussed in more detail in the next section.

#### Convergence of the proposed CORDIC algorithm

The minimum convergence range required for exponential computation is $0\le Z_0\le 1$. Tthe small convergence range of R4HR-CORDIC can be defined as $\vert Z_1 \vert \le \sum _{j=1}^{\infty } \tanh _4^{-1} (2\times 4^(-j))=0.502$. To increase the convergence range to the required value, we propose to rotate the vector through one additional rotation angle, $\tanh _4^{-1}(0.625)$, as follows:

34 $$\begin{aligned} \begin{bmatrix} X^{1} \\ Y^{1} \end{bmatrix}&= \begin{bmatrix} 1 &{} 0.625\sigma _0 \\ 0.625\sigma _0 &{} 1 \end{bmatrix} \begin{bmatrix} X^{0} \\ Y^{0} \end{bmatrix} \nonumber \\ Z^{1}&= Z^0 - \tanh _{4}^{-1}\left( 0.625 \sigma _{0} \right) \end{aligned}$$

where, $\sigma _0=1$ and 0 indicate the rotation of the vector through $\tanh _{4}^{-1}\left( 0.625 \right)$ and no rotation, respectively. This additional rotation has the scale factor $K_0=\sqrt{(1-(\sigma _0 0.6252)^2)}$, which depends on $\sigma _0$. As discussed earlier, in the proposed CORDIC algorithm, the selection function $\sigma _0$ for the first three iterations needs to be pre-computed for scale factor compensation. The parameter $\sigma _0$ of the additional rotation also needs to be pre-computed as it has a variable scale factor. Once the selection functions are known, the scale factor can be pre-computed and stored on a ROM table. Later, $X_0$ is initialized with $\frac{1}{K_h}$, and the scale factor can be compensated without any additional hardware. The concept of high-radix SRT division is used to derive the convergence and selection criteria. The lower (L) and upper (U) limits to select $\sigma _0$, $\sigma _1$, $\sigma _2$, and $\sigma _3$ can be defined as follows:

35 $$\begin{aligned} L&=\tanh _{4}^{-1}\left( 0.625 \sigma _{0} \right) +\tanh _{4}^{-1}\left( \sigma _{1}4^{-1} \right) + \tanh _{4}^{-1}\left( \sigma _{2}4^{-2} \right) +\tanh _{4}^{-1}\left( \sigma _{3}4^{-3} \right) -\frac{2}{3}\tanh _{4}^{-1}\left( 4^{-3} \right) \nonumber \\ U&=\tanh _{4}^{-1}\left( 0.625 \sigma _{0} \right) +\tanh _{4}^{-1}\left( \sigma _{1}4^{-1} \right) + \tanh _{4}^{-1}\left( \sigma _{2}4^{-2} \right) +\tanh _{4}^{-1}\left( \sigma _{3}4^{-3} \right) +\frac{2}{3}\tanh _{4}^{-1}\left( 4^{-3} \right) \end{aligned}$$

The above limits are pre-computed for all possible combinations of $\sigma _0$, $\sigma _1$, $\sigma _2$, and $\sigma _3$ to find the intervals (L, U). Later, the overlapping area between two intervals is found to choose selection criteria. For example, the intervals are [0.4906, 0.5057] and [0.4794, 0.4944] for ($\sigma _0$, $\sigma _1$, $\sigma _2$, and $\sigma _3$)=(0,2,2,1) and (0,2,2,0), respectively. The overlapping between these two intervals is [0.4906, 0.4944]. As a result, any value from this interval can be chosen to select $\sigma _0=0$, $\sigma _1=2$, $\sigma _2=2$, and $\sigma _3=1$. In order to indicate the selection criteria, nine bits must be used for each value of the selection criterion in the proposed method. The criteria to choose $\sigma _0$, $\sigma _1$, $\sigma _2$, and $\sigma _3$ along with the scale factors are listed in the Table 8.

The selection criteria to choose $\sigma _j$ for iterations $j\ge 4$ can be made independent of the iteration index. According to the method^30,33^, we define the new variable $W_j$ as $W_j=4^jZ_j$. The new variable $W_j$ has to be bounded by upper and lower limits. The upper and lower limits of the new variable $W_j$ can be defined as follows:

36 $$\begin{aligned} L_j[q]=4^j\left[ \tanh _4^{-1}(4^{-j}\sigma _{j})-\frac{2}{3}\tanh _4^{-1}(4^{-j})\right] \nonumber \\ U_j[q]=4^j\left[ \tanh _4^{-1}(4^{-j}\sigma _{j})+\frac{2}{3}\tanh _4^{-1}(4^{-j})\right] \end{aligned}$$

Since $L_j[q]$ and $U_j[q]$ are monotonous functions, i.e. $L_j[q] \le L_j[q+1]$ and $U_j[q] \le U_j[q+1]$, the selection criteria can be made independent of iteration index. As a result, the largest value of the lower limit (i.e. $L_{\infty }[q]$) and the smallest value of the upper limit (i.e. $U_4[q]$) are chosen to make selection criteria independent of the iteration index. The selection criteria to choose $\sigma _j$ for iteration index $j\ge 4$ is given below.

37 $$\begin{aligned} \sigma _{j}={\left\{ \begin{array}{ll} 2: \text {if} &{}W_{j}\ge 1.125\\ 1: \text {if} &{} 1.125>W_{j}\ge 0.375\\ 0: \text {if} &{} 0.375>W_{j}\ge -0.375\\ -1: \text {if} &{} -0.375>W_{j}\ge -1.125\\ -2: \text {if} &{} -1.125>W_{j} \end{array}\right. } \end{aligned}$$

In the proposed algorithm, criteria, given in Eq. (37), are used to decide the selection function for any iteration index $j\ge 4$. The selection functions for the first four iterations are pre-computed and stored on a ROM table. The scale factor related to these rotations is defined as $K_h=\sqrt{\left( 1-(0.625\sigma _0)^2\right) \left( 1-\sigma _1^24^{-2j} \right) \left( 1-\sigma _2^24^{-4j} \right) \left( 1-\sigma _3^24^{-6j} \right) }$ This scale factor can be compensated by taking the initial value of the X-coordinate of the rotating vector as $X_0=\frac{1}{K_h}$. The pre-computed scale factor is stored on a ROM table along with a selection function. When $j\ge 4$, the algorithm executes the scaling-free computation. The scale factor compensation for these iterations is not required. The pre-computed scale factors and selection function are accessed from the ROM table by comparing the initial angle $Z_0$ with the selection criteria listed in Table 8. The proposed CORDIC algorithm is summarized in Table 9.

**Table 9:** Computational flow of the R4HR-CORDIC.

Iteration index	Datapath			Operation
	*X*	*Y*	*Z*	
Prescaler	–	–	–	Pre-scale the input angle add selection function
$j=0$	$X_1=\frac{1}{K_h}$	$Y_1=\frac{\sigma _0}{K_h}\left( 2^{-1}+2^{-3}\right)$	$Z_1=Z_0-\tanh _4^-1(0.625\sigma _0)$	Compute $X_1$, $Y_1$, and $Z_{1}$ based on $\sigma _0$.
$1\le j \le 3$	$X_{j+1}=X_j+\sigma _j4^{-j}Y_j$	$Y_{j+1}=Y_j+\sigma _j4^{-j}X_j$	$Z_{j+1}=Z_j-\tanh _4^{-1}(\sigma _j4^{-j})$	Compute conventional radix-4 hyperbolic rotation
$4\le j \le 6$	$X_{j+1}=X_j \left( 1+\frac{\sigma _j^24^{-2j}}{2} \right)$ $+Y_j\left( \sigma _j4^{-j}+\frac{\sigma _j^34^{-3j}}{8}\right)$	$Y_{j+1}=Y_j \left( 1+\frac{\sigma _j^24^{-2j}}{2} \right)$ $+X_j\left( \sigma _j4^{-j}+\frac{\sigma _j^34^{-3j}}{8}\right)$	$Z_{j+1}=Z_j -\tanh _4^{-1}\left( \frac{\sigma _j4^{-j}+\frac{\sigma _j^34^{-3j}}{8}}{ 1+\frac{\sigma _j^24^{-2j}}{2} } \right)$	Compute scaling-free hyperbolic rotation with two terms of hyperbolic sine and cosine.
$7\le j \le 12$	$X_{j+1}=X_j \left( 1+\frac{\sigma _j^24^{-2j}}{2} \right)$ $Y_j\left( \sigma _j4^{-j}\right)$	$Y_{j+1}=Y_j \left( 1+\frac{\sigma _j^24^{-2j}}{2} \right)$ $X_j\left( \sigma _j4^{-j}\right)$	$Z_{j+1}=Z_j -\tanh _4^{-1}\left( \frac{\sigma _j4^{-j}}{1+\frac{\sigma _j^24^{-2j}}{2}} \right)$	Compute scaling-free hyperbolic rotation with two terms of hyperbolic cosine and one term of hyperbolic sine.
$13\le j \le \frac{n}{2}$	$X_{j+1}=X_j+ Y_j\left( \sigma _j4^{-j} \right)$	$Y_{j+1}=X_j+ X_j\left( \sigma _j4^{-j} \right)$	$Z_{j+1}=Z_j -\tanh _4^{-1}\left( \sigma _j4^{-j}\right)$	Compute scaling-free hyperbolic rotation with one term of hyperbolic sine and cosine.

Table 9 provides the operations carried out by the various rotators in each stage. In the pre-processing stage, the integer and fraction parts of the input angle are derived. In this stage, pre-computed scale factor, and selection functions are retrieved from the ROM table. In the next stage, $X_1$ is initialized with a pre-computed scale factor, and $Y_1$ is computed using the relation given in Table 9. The* Z* rotator of this stage rotates the two-dimensional vector by an angle $\tanh _4^{-1}(0.625)$ if $\sigma _0=1$. It does not perform the rotation otherwise. The next three stages compute the standard radix-4 HR CORDIC iterations based on the $\sigma _j$ received from the previous stage. The next three stages compute scaling-free iterations wherein hyperbolic sine and cosine are approximated using two terms. In Taylor’s approximation of hyperbolic sine, 3! is replaced with $8(2^3)$ so that computation can be achieved using binary shift only. The absolute error introduced by this approximation is $1.4 \times 10^{-17}$ for $j=4$ and $\sigma _4=2$. The second term in the Taylor approximation of hyperbolic sine and cosine can be ignored for the iterations $7 \le j\le 12$ and $13 \le j\le \frac{n}{2}$. As a result, the remaining stages compute the standard radix-4 HR CORDIC for $j\ge 13$. The architecture and hardware required to compute these iterations are discussed in the next section.

**Figure 5:**
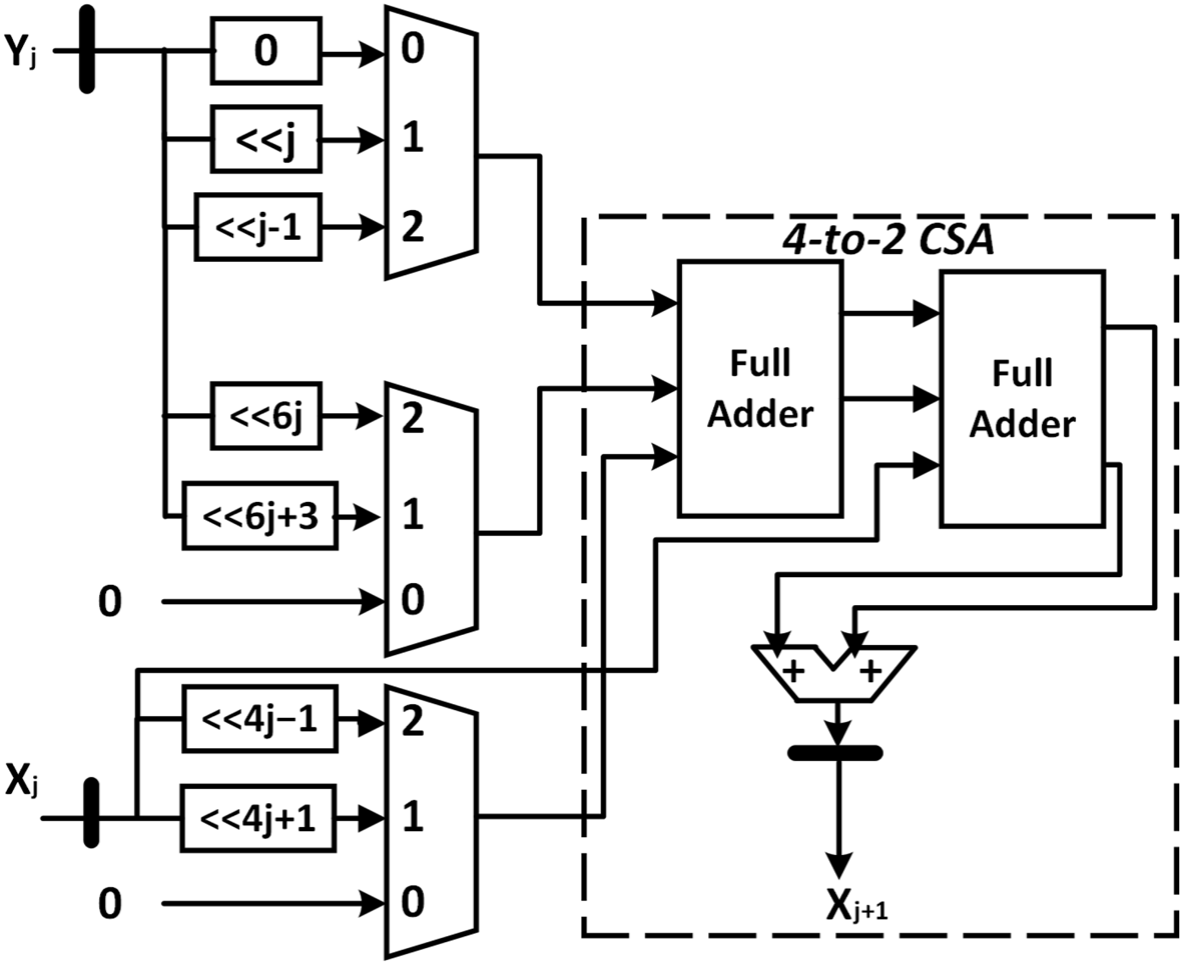
Architecture of R4HR-CORDIC stage with $4\le j \le 6$.

**Figure 6:**
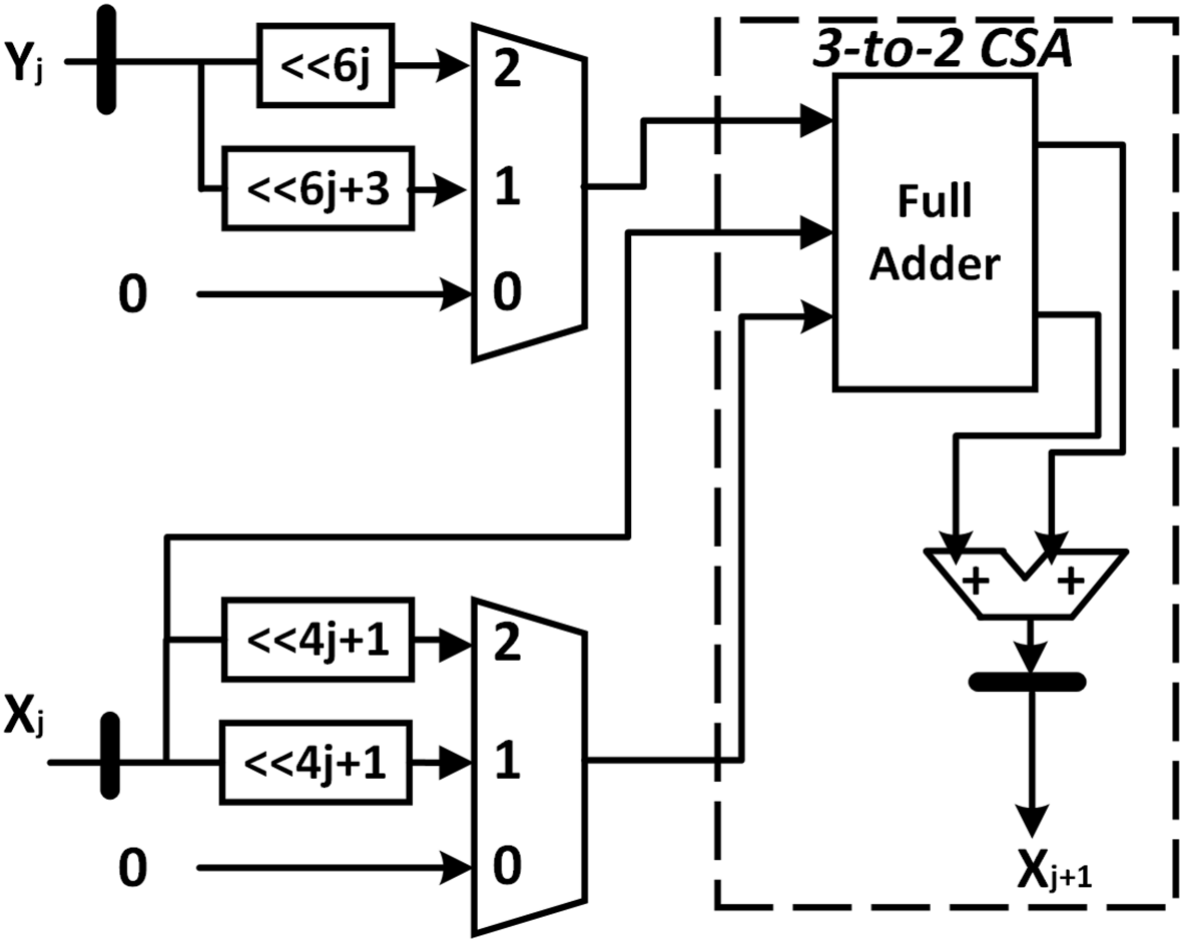
Architecture of R4HR-CORDIC stage with $7\le j \le 12$.

#### The architecture of the proposed CORDIC algorithm

The architecture, timing analysis, and hardware complexity of the proposed CORDIC algorithm are discussed in this section. The first stage of the proposed algorithm is the normalizer that separates the integer ($V_I$) and fractional ($V_Z$) parts of the input angle. At the end of the computation, the result is shifted by $2V_{I}$-bits to achieve the actual results. This stage accesses the scale factor and selection functions from the ROM table based on the value of the input angle after the normalization. The* X* rotator of this stage is simple, and it only initializes the $X_1$ using $\frac{1}{K_h}$. The* Y*-rotator computes the $\frac{0.625}{K_h}$ by adding two partial products $\frac{0.5}{K_h}$ and $\frac{0.125}{K_h}$ using adder. If the selection function $\sigma _{0}$ is zero, $Y_1$ will be initialized with a zero to indicate that no rotation has occurred. If $\sigma _{0}$ is one, $Y_1$ is set to $\frac{0.625}{K_h}$. Similarly, the Z rotator computes $Z_1$ based on the value of $\sigma _0$. The critical path delay of* Y* and* Z* rotators are equal, and it is given as $T_0=T_{ROM}+T_{MUX21}+T_{ADD}$ where,$T_{ROM}$ is a delay of read-only memory (ROM), $T_{MUX21}$ is the delay of 2-to-1 multiplexer, and $T_{ADD}$ indicates the delay of the adder.

Since fixed-point representation is used, the normalizer computes the normalized value by shifting the input angle using fixed bits and it does not add delay. The VLSI implementation of this stage requires two adders and two 2-to-1 multiplexers each for* Y* and* Z* rotators. As discussed in the section on word length analysis, the scale factor is represented using 30-bit, and selection functions $\sigma _0$ and $\sigma _1$ to $\sigma _3$ can be represented using one and three bits, respectively. As a result, the ROM table size of this stage is 90x40 bits. The next three stages compute the standard radix-4 HR CORDIC iterations based on the pre-computed selection function received from the previous stage. The architecture of the* X* and* Y* rotators of these stages is similar to the architecture of the R4HV-CORDIC. The VLSI implementation of this stage requires three 3-to-1 multiplexers and three adders. The critical path delay of this stage includes the delay of the adder ($T_{ADD}$) and 3-to-1 multiplexer ($T_{MUX31}$), and it is given as $T_0=T_{MUX31}+T_{ADD}$. The total hardware complexity of this state is three 3-to-1 multiplexers and three adders.

The next three stages compute the scaling-free iterations. These stages use two terms of the Taylor approximation of hyperbolic sine and cosine to make computation scaling-free. The architecture of the* X* and* Y* rotators is similar and only the architecture of the X rotator is shown in Fig. 5. All the terms of the Taylor approximation can be multiplied with $X_j$ and $Y_j$ using binary shift only and it can be implemented using only a 3-to-1 multiplexer. For example, for iteration index* j*=4, the term $\frac{\sigma _j^24^{-2j}}{2}$ in cosine approximation can be simplified to $2^{-15}$, $2^{-17}$, and 0 for $\sigma _4=2$, 1, and 0, respectively which can be implemented using a 3-to-1 multiplexer. The X and Y datapath of these stages requires three 3-to-1 multiplexers to generate three partial products and a 4-to-2 carry-save adder (CSA) to add four partial products as shown in Fig. 5. The VLSI implementation of a 4-to-2 CSA requires two full adders and one adder. The Z rotator of this stage compares $Z_j$ with the criteria given in Table 8 to derive the $\sigma _j$. Later, $Z_{j+1}$ is computed based on the value of $\sigma _j$ by adding or subtracting the $\tanh _4 \left( \sigma _j4^{-j}\right)$ from $Z_j$. The critical path delay of the X and Y rotators of this stage is dominant compared to the Z rotator and it is given as $T_{2} = T_{COMP}+T_{ADD}+2T_{FA}+T_{MUX31}$, where, $T_{COMP}$ indicates the delay of the comparator, and $T_{FA}$ is the delay of full adder. The timing and hardware complexity of these stages is the highest compared to other stages. The VLSI implementation of this stage requires seven 3-to-1 multiplexers, four full adders, and three adders.

The stages with iteration index $7\le j\le 12$, perform the scaling-free rotation with one term and two terms of the Taylor approximation of sine and cosine, respectively. As shown in Fig. 6, the VLSI implementation of this stage uses five 3-to-1 multiplexers, two full adders, and three adders. The delay of this computation is equal to $T_{3} = T_{COMP}+T_{ADD}+T_{FA}+T_{MUX31}$. The scale factor can be assumed one for the remaining stages. For example, for j=13, the absolute error is $4.44\times 10^{-15}$ if the scale factor is assumed one. The VLSI implementation of the rest of the stages requires three adders and three 3-to-1 multiplexers. The modified R4-HR CORDIC has a total hardware complexity of 84 adders and 62 3-to-1 multiplexers for 40-bit precision, which is a reasonable improvement from the 126 adders of the radix-2 CORDIC algorithm.

## Experimental results and discussion

We give the experimental data from our research study in this section and provide an in-depth evaluation of the outcomes.

### Datawidth analysis

**Table 10:** Datawidth analysis.

Task	$P^N$				$P^{\dfrac{1}{N}}$			
	Operation	Variable	Data format	Word length	Operation	Variable	Data format	Word length
Logarithm	Normalizer	*P*	FXP(8,27)	35	Normalizer	*P*	FXP(21,27)	48
	R4HV	*p*	FXP(4,31)	35	R4HV	*p*	FXP(4,44)	48
	Adder	q, $\log _24p$	FXP(4,27)	31	Adder	q, $\log _4p$	FXP(5,27)	32
Division / Multiplication	Multiplier	$\log _4(P)$, N	FXP(5,27)	32	R4LV	$\log _4(P)$, N	FXP(11,27)	38
	Normalizer	$\log _{4}(P){N}$	FXP(3,27)	30	Normalizer	$\dfrac{\log _4(P)}{N}$	FXP(4,27)	31
Exponential	R4HR	*q*, $\log _{4}(P){N}$	FXP(3,27)	30	R4HR	*q*, $\dfrac{\log _{4}(P)}{N}$	FXP(3,27)	30
	Adder	$\cosh (.)$, $\sinh (.)$	FXP(3,27)	30	Adder	$\cosh (.)$, $\sinh (.)$	FXP(3,27)	30

The data width of the* X*,* Y*, and* Z* rotators is a crucial factor to consider before implementing the hardware of the proposed methodology. The data width determines the number of bits used to represent the input and output variables of the various stages of the proposed method. The fixed-point representation is used to represent the variables. The format FXP(a,b) indicates a-1 integer bits, b fraction bits, and one sign bit are used to represent the number using fixed-point. As per the methodology presented in^25,26^, the input range of P is assumed to be $P \in \left[ 10^{-6},10^{6} \right]$ and $N \in \left[ 2, 1002\right]$. For comparison purposes, 27 bits are used to represent the fractional part of the input number P. The maximum value of P is $10^{6}$, which can be represented using 20 integer bits. As a result, the total number of binary bits required to represent the P is 48 (FXP(21, 27)).

The first step in the logarithm computation is the normalizer. The convergence range of the proposed radix-4 HV CORDIC algorithm is $p = \left[ \frac{1}{4.19},4.19 \right]$. The normalization process in the proposed method rearranges the fixed-point representation of the input number P as FXP(4, 44) to bring down the P into the convergence range of the proposed R4HV-CORDIC. As a result, FXP(4, 44) precision is used to represent the* X* and* Y* coordinates of the R4HV-CORDIC algorithm. The integer data width required to represent the Z rotator of the R4HV-CORDIC algorithm depends on the value of $\log _4(P_{max})$. Since the maximum value of P is $10^{6}$, four integer bits are required to represent the logarithm of the maximum of P. Also, the normalization factor (q) is 9 for the $P_{max}$. Hence, FXP(5,27) precision is considered to represent q, and $\log _4(P_{max})$.

The number of bits required to represent the integer part of the input of R4LV-CORDIC depends on the maximum value of N. Since $N_{max} = 1002$, ten bits are required to represent the integer part of N. As a result, the FXP(11,27) precision is used to represent the N, and $\log _4{P_{max}}$ is extended to the same precision. The maximum input to R4HR-CORDIC is $Z_0=\frac{\log _4(2^{20})}{2}=5$. Three bits are required to represent the integer part of the $Z_0$ of R4HR-CORDIC. As a result, FXP(4,27) precision is taken to represent the $Z_0$. After the normalization process, the R4HR-CORDIC only rotates the vector through the fractional part of the $\frac{\log _4(P)}{2} \approx 1$ and since cosh4(1) = 2.125, the integer part of the* X* and* Y* inputs of the R4HR-CORDIC are represented using two bits. The final output is shifted by $Z_I$ bits to get an actual exponential value. Additional 9 bits are considered to represent the factional part of the* X* and* Y* inputs. Hence, FXP(3, 36) precision is considered to represent the* X* and* Y* inputs.

Next, we analyze the data width required for the computation of $P^N$. As given in^26^, we assume the range of P and N to be limited to the interval $\left[ 10^{-2},10^2 \right]$ and $\left[ 1, 5 \right]$, respectively. The maximum input to R4HV-CORDIC is 100. Hence, 7-bit is considered to define the integer part of the input* P*. For an average precision of $10^{-7}$, 27-bit is used for the fraction part of the input P. As a result, in the proposed methodology, input P is represented using the precision FXP(8,27). The first step in the logarithm computation is pre-log normalization. After the normalization process, normalized* P* (p) can be defined with the precision FXP(4,31). The maximum output of the logarithm computation is $log_4(100)=3.32$, and hence, 2-bit is considered to represent the integer part of $log_4(100)$. Hence, *Z*-datapath and $log_4(p)$ are defined using precision FXP (3,27).

The input to the multiplier is $log_4(p)$ and N. The integer part of the output of the multiplier can be represented using 5 bits as $\log _4(p)\times N_{max}=16.61$. Hence the output of the multiplier is defined using precision FXP (6,27). The next step is to calculate the exponential. The first step in an exponential calculation is normalization. In the normalization process, the integer and fractional parts of the input angle of radix-4 HR CORDIC are separated. The fractional part of the input angle can be represented using FXP(3,27). As discussed earlier, the integer part of the X and Y data paths is represented using 2 bits. Hence, the X and Y inputs of the R4HR-CORDIC are represented using FXP (3,27). The data width required to represent the various variables at different stages is summarized in Table 10.

### Accuracy and number of iterations

**Figure 7:**
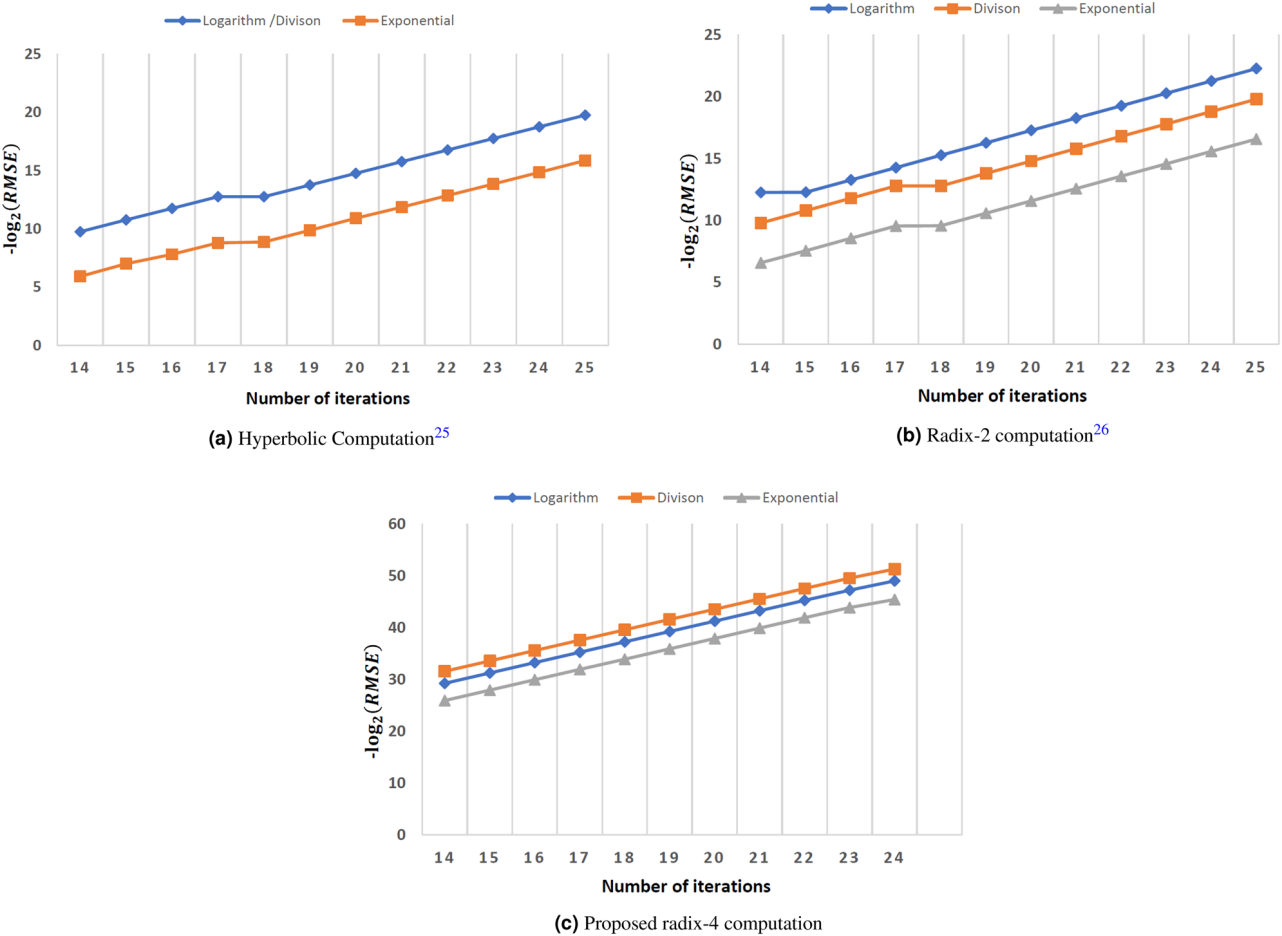
Error analysis.

To verify the proposed methodology, RMSE, and maximum absolute error (max(AE)) is measured using the Eq. (38).

38 $$\begin{aligned} RMSE&= \sqrt{\dfrac{\sum _{i=1}^{S}\left( A_i - B_i\right) ^2}{S}} \nonumber \\ max(AE)&=max \left( \left| \dfrac{A-B}{A} \right| \right) \end{aligned}$$

where $A_i$ and $B_i$ indicate the actual and calculated values. The desired accuracy or the number of iterations may be changed to modify the precision of the output coordinates in the conventional CORDIC algorithm. The accuracy of the coordinates increases with the number of executed iterations. However, adding the iterations increases the cost of the computation. Also, the high-radix CORDIC algorithm achieves the desired accuracy faster than the conventional CORDIC algorithm. The study should be carried out to see the impact of iterations on accuracy for various approaches. Figure 7 demonstrates the plot between the iterations performed and the accuracy for different approaches. The accuracy is measured by locating RMSE in output coordinates using equation $-\log _2{(RMSE)}$. Figure 7a–c demonstrate the iteration versus accuracy graphs for hyperbolic rotation, base-2 rotation, and proposed rotation. From this Fig. 7, it can be concluded that the proposed method achieves high accuracy compared to other approaches for the same number of iterations. However, the hardware required to carry out the radix-4 CORDIC rotation is slightly higher than the standard radix-2 CORDIC. The hardware required to implement the proposed approach is analyzed in the next section.

**Table 11:** Error performance.

Stages	^25^		^26^		Stages	Proposed	
	RMSE	Max(AE)	RMSE	Max(AE)		RMSE	Max(AE)
16	9.73E-04	7.98E-03	7.06E-04	3.80E-03	8	4.66E-04	2.76E-03
20	1.00E-04	8.70E-04	4.40E-05	2.27E-04	10	2.92E-05	1.70E-04
24	6.25E-06	4.97E-05	2.76E-06	1.49E-05	12	1.82E-06	1.03E-05
28	3.89E-07	3.21E-06	1.72E-07	9.02E-07	14	1.14E-07	6.69E-07
32	2.43E-08	2.14E-07	1.08E-08	5.68E-08	16	7.12E-09	4.13E-08
36	1.52E-09	1.33E-08	6.72E-10	3.54E-09	18	4.45E-10	2.59E-09

**Table 12:** Hardware complexity comparison to compute $P^{1/N}$.

Computation	^25^			^26^			Proposed		
	Stages	TC	RMSE	Stages	TC	RMSE	Stages	TC	RMSE
Log	25	182016	1.10E-06	22	155160	1.59E-06	10	102156	4.01E-07
Division	23	83904	1.12E-06	23	83904	1.15E-06	11	68288	1.11E-06
Exponentials	25	146952	1.67E-05	22	95760	1.29E-05	11	89340	1.21E-05
% Improvement	37.07			22.41

**Table 13:** Hardware complexity comparison to compute $P^{N}$.

Computation	^25^			^26^			Proposed		
	Stages	TC	RMSE	Stages	TC	RMSE	Stages	TC	RMSE
Log	24	126000	2.44E-06	22	113552	1.15E-06	10	78600	3.07E-07
Multiplier	1	31482	3.66E-06	1	31482	1.72E-06	1	31482	4.61E-07
Exponentials	27	257424	6.17E-05	22	95760	2.72E-05	11	89340	1.17E-05
% Improvement	51.94			17.18

**Table 14:** Hardware complexity comparison to compute $P^{1/N}$.

Computation	^25^			^26^			Proposed		
	Stages	TC	Accuracy	Stages	TC	Accuracy	Stages	TC	Accuracy
Log	48	340992	2.63E-13	45	313344	3.78E-13	20	195276	3.82E-13
Division	38	138624	3.36E-11	38	138624	3.36E-11	19	117952	1.67E-11
Exponentials	39	225576	6.37E-10	38	208848	3.32E-10	19	170100	3.11E-10
% Improvement	31.46			26.86

**Table 15:** Hardware complexity comparison to compute $P^{N}$.

Computation	^25^			^26^			Proposed		
	Stages	TC	Accuracy	Stages	TC	Accuracy	Stages	TC	Accuracy
Log	37	193200	3.23E-10	35	178920	1.53E-10	15	158028	3.06E-10
Multiplication	1	31482	4.85E-10	1	31482	2.29E-10	1	31482	4.59E-10
Exponentials	36	196128	1.63E-08	32	166752	2.78E-08	15	137484	1.48E-08
% Improvement	22.29			13.29					

To measure the error performance, a total of $10^5$ samples of input P are generated in the range from $10^{-6}$ to $10^6$ using the logarithm step to cover the entire range with the minimum samples. The error is measured for the $N{{\rm th}}$ root computation with* N*=5. Table 11 compares the RMSE and max(AE) for the proposed method and approaches presented in^25^ and ^26^. The number of stages used to measure the error is also mentioned in Table 11. Hyperbolic and binary logarithmic CORDIC repeats the iteration with indexes j=4, 13, 40, ... and repeated iteration is considered as a stage in Table 11. For example, approach^26^ goes through the iterations with indexes j=1 to 14, and iterations 4 and 13 are repeated, resulting in a total of 16 iterations (stages). However, the proposed method uses radix-4 computations where iterations are not required to be repeated. Hence, the proposed approach goes through the iterations j=1 to 8 resulting in 8 iterations (stages). From Table 11, it is apparent that the proposed algorithm uses half the stages compared to approaches^25^ and^26^ and has better error performance. Next, the hardware complexity of the proposed approach is compared with approaches^25,26^, and it is discussed next.

### Hardware complexity analysis

Hardware analysis is carried out quantitatively by computing the transistors required to implement the proposed architecture for two configurations. In the first configuration, the number of iterations of each CORDIC configuration is considered as given in^25,26^. As discussed earlier, the standard CORDIC produces 1-bit precision in each iteration, whereas the radix-4 CORDIC generates 2-bit precision. Hence, in the second configuration, we chose the number of iterations based on the data width of the different variables of three CORDIC configurations.

Adder/subtractor, multiplexer, ROM, and comparator are the basic building block of the proposed algorithm. In the proposed design R4HV, R4LV, and R4HR CORDICs compute logarithm $(\log _4(P))$, divison $\left( \log _4(P)/N \right)$ and exponential $4^{\left( \log _4(P)/N \right) }$, respectively. For b-bit datapath, 24b and 48b transistors are required to implement the simple adder and adder/subtractor, respectively^25,26^. Similarly, 6b, 10b, and 24b transistors are required to implement b-bit ROM, multiplexer, and comparator^34^. Each stage of the proposed R4HV CORDIC uses one adder/ subtractor and multiplexer for each X, Y, and Z datapath resulting in 174b transistors. Along with that, R4HV uses 1250 bits of memory to store pre-computed selection functions and criteria. Except for the first two stages, all the stages of R4HV use 8-bit comparators. Hence, the transistors required to implement R4HV are summed up in the below equation.

39 $$\begin{aligned} TC_{R4HV}=174nb+960(n-2)+10956 \end{aligned}$$

where n indicates the number of stages. Similarly, each stage of the R4LV CORDIC requires an adder/subtractor and multiplexer each for the Y and Z datapaths resulting in 116bn transistors. However, the calculation of transistors needed to implement R4HR CORDIC depends on the target precision, and based on the precision, we consider the order of the Taylor series approximation. The first two stages of the R4HR CORDIC require two simple adders. The following three stages use the precomputed selection function, and hardware complexity is the same as the R4HV CORDIC. The rest of the stages perform the scaling-free computation that requires seven adders and five multiplexers. R4HV stores precomputed scale-factor and selection functions on a memory of 49x90 bits. A total of $2096b+26460$ transistors are needed to implement R4HR CORDIC. Using these illustrations, transistors needed to implement log, division/multiplication, and exponentials are listed in Tables 12 and 13 for the first configuration to compute the $N{{\rm th}}$ root and power. Similarly, Tables 14 and 15 summarize the transistors for the second configuration. The RMSE achieved by each computation is also mentioned in the Tables. From Table 12, it is apparent that the proposed $N{{\rm th}}$ root implementation has $37\%$ and $22\%$ less hardware utilization than approaches^25^ and^26^, respectively. Table 13 shows that the suggested $N{{\rm th}}$ power computation uses hardware $51\%$ and $17\%$ less than approaches^25^ and^26^.

**Table 16:** FPGA implementation.

Implementation	$R^{\dfrac{1}{N}}$		$R^N$	
	Slice LUTs	%Improvement	Slice LUTs	%Improvement
^25^	17684	47.27	17303	52.64
^26^	14706	36.60	12416	34.00
Proposed	9324		8195	

The proposed design is implemented on FPGA Virtex-6 to check the actual hardware utilization. Table 16 summarizes the resource utilization in terms of slice LUTs for approaches^25^ and^26^ and the proposed design for root and power computations. It is apparent from Table 16 that the proposed implementation has used $47\%$ and $36\%$ less FPGA resources than^25^ and^26^ for root computation. Further, the proposed implementation has $52\%$ and $34\%$ less FPGA resources than^25^ and^26^ for power computation. Multiplexers and ROM are the additional resources required to implement the proposed design. The FPGA implements these components more efficiently. For example, LUT6 can work as a 32-bit distributed ROM and adder/subtractor, and a simple adder consumes similar hardware.

## Conclusion

The computation of $N{{\rm th}}$ root and $N{{\rm th}}$ power plays a crucial role in many real-time applications. These functions help provide valuable solutions to complex equations. The real-time hardware implementation of such functions demands a high clock rate with less hardware utilization. The Newton-Raphson-based method is a traditional way to compute such functions. However, a real-time realization of these methods consumes a lot of hardware resulting in a slow clock rate. Another way to implement these functions is to use various CORDIC configurations to compute mathematical operations. However, the standard CORDIC algorithm suffers from the iterative process. In the proposed method, we have used the various radix-4-based CORDIC configuration to compute log, division/multiplication, and exponentials to implement $N{{\rm th}}$ root and power computations. The main objective of the proposed work is to carry out the FPGA implementation. Therefore, we have conducted a qualitative analysis and FPGA implementation of the proposed approach. The quantitative analysis suggests that the proposed $N{{\rm th}}$ root implementation has $37\%$ and $22\%$ less hardware utilization than approaches^25^ and^26^, respectively. The FPGA implementation indicates that the proposed method has $36\%$ and $34\%$ less hardware utilization than the recent approach^26^ for root and power computations, respectively. We decided to begin with FPGA implementation due to its quick implementation and validation capabilities. This approach allows us to validate our design and make necessary improvements before the ASIC implementation. However, we will carry out the ASIC implementation of the proposed methodology using commercial CMOS libraries in the future.

## Data Availability

The datasets used and/or analysed during the current study available from the corresponding author on reasonable request.
